# Cu-metal organic frameworks (Cu-MOF) as an environment-friendly and economical catalyst for one pot synthesis of tacrine derivatives†

**DOI:** 10.1039/c9ra10111j

**Published:** 2020-01-09

**Authors:** Hoda Mollabagher, Salman Taheri, Mohammad majid Mojtahedi, SeyedAmirhossein Seyedmousavi

**Affiliations:** Chemistry and Chemical Engineering Research Center of Iran PO Box 14115-186 Tehran Iran taheri@ccerci.ac.ir; Process Engineering Department, Faculty of Chemical Engineering, Tarbiat Modares University Tehran Iran

## Abstract

The present work describes the catalytic activity of Cu-MOF for the one-pot synthesis of tacrine derivatives *via* a four-component reaction of 2-hydroxynaphthalene-1,4-dione, aldehydes, malononitrile and cycloketones in the presence of AlCl_3_. The structure of the synthesized compound is confirmed by ^1^H NMR, ^13^C NMR, IR, and MASS. The catalyst prepared under pressure is characterized by powder X-ray diffraction and SEM. The noteworthy advantages of this procedure include its broad substrate scope, high yields up to 93%, atom economy, using readily available starting materials and a powerful recyclable nano catalyst. Additionally, there is no need to use column chromatography for purifying products so, it has the potential for large-scale applications in pharmaceutical industries. Another advantage of this method is the ability to recycle the catalyst up to 3 times and reuse it.

## Abstract 2

The present work describes the catalytic activity of Cu-MOF for the one-pot synthesis of tacrine derivatives *via* a four-component reaction of 2-hydroxynaphthalene-1,4-dione, aldehydes, malononitrile and cycloketones in the presence of AlCl_3_.

## Graphical abstract

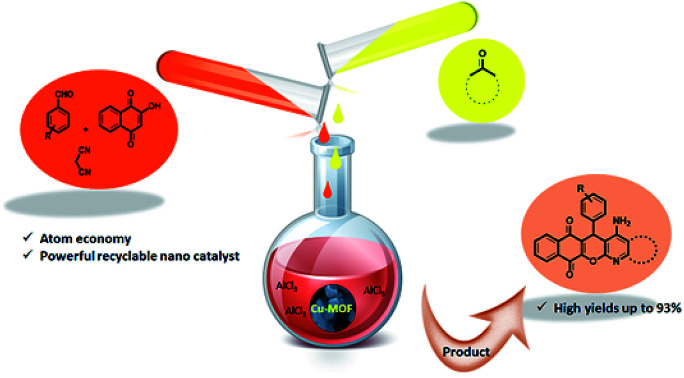

## 1. Introduction

According to the World Health Organization, over 30 million people worldwide suffer from Alzheimer's disease (AD), and research has shown that this number is rising substantially.^1^ AD is actually a type of brain dysfunction that gradually degrades the mental abilities of the patient and leads to memory impairment and dementia. Acetylcholine is one of the substances that transmits the message between the two nerve cells and then decomposes with cholinesterase. In AD reducing the acetylcholine disrupts the transmission of messages between the two nerve cells.^2^ Therefore, many studies have been done to strengthen the cholinesterase system includes acetylcholine release agents such as 4-aminopyridine (1), and anticholinesterase drugs, such as physostigmine (2) and acetylcholinesterase inhibitors like tacrine (3) and 4-aminoquinoline (4).
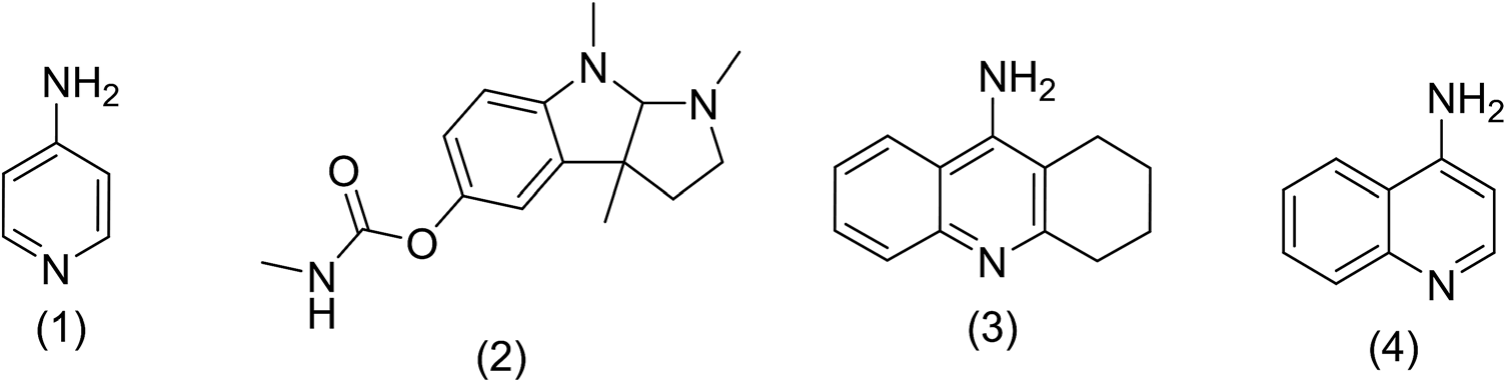


Among the listed compounds, tacrine is one of the drugs that boost acetylcholine by inhibiting cholinesterase enzymes^3^ and is known as an important reference with amazing pharmacological properties. It was first identified in 1993 as a drug for the treatment of AD.^4^ Studying of tacrine analogues is still of interest to researchers investigating AD. As a result, several methods have been investigated for the synthesis of tacrine and their analogues. Recently, synthesis of tacrine analogues with modern strategies including high efficiency and short time is a great interest of chemists and pharmacists.^5,6^ Synthesis of tacrine analogues was mostly reported in the presence of a Lewis acid such as AlCl_3_,^7^ ZnCl_2_,^8^ BF_3_/Et_2_O,^9^ silica gel/*p*-toluenesulfonic acid.^10^ Khalilzadeh and coworkers reported new method for the preparation of tacrine analogues *via* applying microwave irradiation by using silica gel/*p*-toluenesulfonic acid as a catalyst to improve reaction time.^10^ However, the main problem with their work refers to microwave irradiation that microwave energy absorbing is possible only to a limited thickness of materials and can be changed due to material absorption and is not easy and precisely applicable for industrial production.^11^

Several reports have been made to design tacrine analogues, including the replacement or hetero-annulated of the benzene ring with the heterocyclic systems. The presence of aromatic or heteroaromatic rings in the tacrine scaffolds results in additional π–π interaction in the structure and have pharmacophoric moiety that can be used for the design of new AChE inhibitors.^12^

Polyfunctionalized 2-amino-3-cyano-4*H*-pyrans are well-known compounds that reactivity has been extensively investigated and used as intermediates in the tacrine synthesis. In this way, it is prepared from Friedländer annulation involving the condensation of 2-amino-3-cyano-4*H*-pyrans with carbonyl compound that contains reactive a-methylene group.^7^ All reported tacrine synthesis procedures from pyranic intermediate, were contained two or more steps.^13^

One-pot synthesis of materials is a simple, facile and effective method in synthetic chemistry. Minimizing the number of steps in the synthesis procedure in order to obtain target compounds is of great interest in the production of chemical and pharmaceutical compounds. The requirement for the rapid and selective structure of biologically active materials for drug discovery has led to the widespread development of heterogenous catalysis, which allows the researcher to reduce the steps of the reactions.^14–17^

Heterogeneous catalysts have also many other advantages such as easily isolation from the mixture of the reaction, be recyclable and less contamination in final product.^18^ Metal organic frameworks (MOFs) attracted the attention of the scientific community around 1990 (ref. 19) and then it has been effectively used as heterogeneous catalysis improving efficiency and selectivity of one-pot reactions. The specific porous structure of MOF containing organic and inorganic active sites is a useful and effective alternative to heterogeneous catalysts.

In recent years, MOFs are highlighted due to its bicompositional nature contains metallic ions and organic ligands together in one complex. Organic ligands and metallic ions could be changed to achieve different efficiencies and yields. Also, the high porosity of these structures has made them a powerful nano-reactor for chemical reactions.^20^ According to the above, the flexibility, physical, chemical, biological properties of these compounds can be very diverse and unique.

Among this kind of materials copper-based MOFs used as heterogeneous acid in the synthesis of the organic compound. Due to the catalytic behavior of MOFs, they have been used in various reactions including the oxidation of benzoquinones, cyanosilylation of aldehydes, and Knoevenagel condensations.

**Scheme 1:**
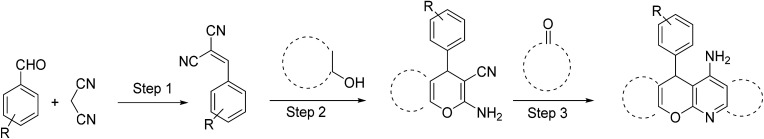
Synthesis of tacrine derivatives from pyranic intermediate.

Up to now most of the reported procedures for tacrine synthesis go through pyranic intermediate which should be separated and purified for the next step of the reaction (Scheme 1).^21,22^

Our work has presented a novel procedure to the synthesis of tacrine derivatives, starting from aldehyde, malononitrile, 2-hydroxynaphthalene-1,4-dione and cycloketone derivative as one-pot reaction without the requirement for separating the pyranic intermediate. Applying the Cu-MOF as heterogeneous catalyst assistances the formation of pyranic intermediates, followed by the addition of aluminum chloride to the Friedländer quinoline reaction, without interfering with the two catalysts in the reaction process. The presence of Cu active sites in Cu-MOF has made it a suitable candidate for the synthesis of pyrene compounds. We describe convenient synthesis methods tacrine derivatives from the simple starting material.

## 2. Experimental

### 2.1. Chemicals and apparatus

All starting materials were purchased from Sigma-Aldrich and Merck companies and used without additional purification. Reactions were monitored by aluminum TLC plate, silica gel coated with fluorescent indicator F_254_. Melting points were measured using a Buchi B-545 apparatus through the capillary tube method and reported without any correction. IR spectra were recorded from KBr disks using FT-IR Bruker Vector-22 infrared spectrometer in the range of 400–4000 cm^−1^. The ^1^H NMR and ^13^C NMR were run on an FT-NMR Bruker UltraShield™ (500 MHz) in DMSO-*d*_6_ and CDCl_3_ as a solvent and the chemical shifts are expressed as *δ* units with tetramethylsilane (TMS) as internal standard. Mass spectra were obtained on an Agilent Technologies apparatus at ionization potential of 70 eV. The concentration of copper in the prepared catalysts was determined using inductively coupled plasma optical emission spectroscopy by SPECTRO ARCOS ICP-OES spectrometer.

### 2.2. General procedure for the preparation of compounds 6a–k

A mixture of 2-hydroxy-1,4-naphthoquinone 1 (0.174 g, 1.0 mmol), aromatic aldehyde 2 (1.0 mmol), malononitrile 3 (0.06 g, 1.0 mmol), and catalytically amount of Cu-MOF (0.02 g) in dry 1,2-dichloroethane (DCE) (5.0 mL) was stirred under reflux condition until completion reaction and disappearance of the starting materials on TLC (EtOAc : *n*-hexane 1 : 1). Then, AlCl_3_ (0.1995 g, 1.5 mmol) and cycloketone 5 (1.5 mmol) were added into the mixture of the reaction. The mixture was continuously stirred under argon atmosphere under reflux. After completion, the mixture was centrifuged to isolated catalyst and then the solvent was evaporated under reduced pressure, H_2_O and THF (1 : 1) were added and the mixture was basified with 10% sodium hydroxide solution to pH = 8–9. After stirring for 30 min, the mixture was diluted using CH_2_Cl_2_ and the organic layer was dried over MgSO_4_. In the end, the crude is crystallization by ethyl acetate to obtain the pure product.

### 2.3. Preparation of nano catalyst Cu-MOF

The nano catalyst was synthesized with modification according to the method described in previous articles.^23^ 0.24 gram (1 mmol) of copper(ii) nitrate trihydrate and 0.24 gram (1 mmol) 1,4-benzenedioic acid gradually dissolve in 20 mL *N*,*N*-dimethylformamide (DMF). The resulting mixture was then transferred into a 25 mL autoclave reactor and placed under 5 barr at 80 °C for 4 hours. After cooling to the room temperature, the precipitate was centrifuged and Cu-MOF turquoise powder was obtained. To activate the catalyst and remove the DMF from the cavities, Cu-MOF was put into Soxhlet extractor for 1 day with dichloromethane as solvent.

The point to consider is that the use of subterranean radiation, as well as reacting in a reactor under pressure, can reduce the synthesis time of MOF from 24 hours to 4 hours, as well as lower the temperature from 100 °C to 80 °C than that of other papers. The ICP-OES showed the Cu content in Cu-BDC structure was about 38.95%.

## 3. Results and discussion

### 3.1. Structural analysis of Cu-MOF nano catalyst

**Fig. 1:**
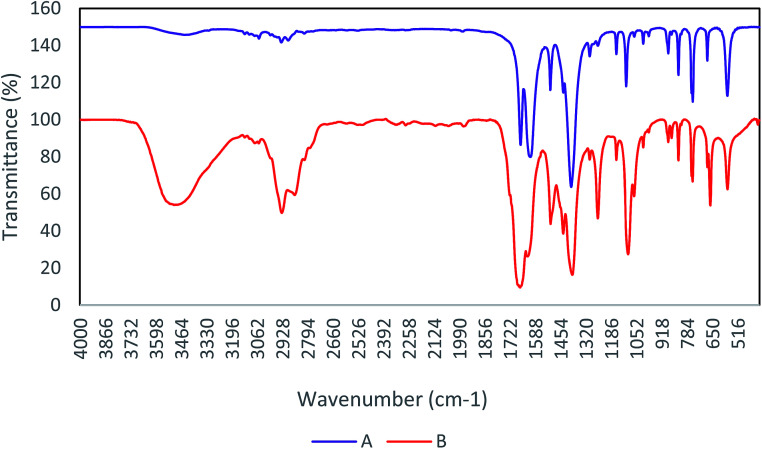
FT-IR spectra of Cu-MOF (A) after activation, (B) before activation that present omitting DMF.

FT-IR is a main technique for determination of the functional groups in the structure of compounds. Therefore, the study of FT-IR spectra is an accepted method among chemists to identify materials.^24,25^ In Fig. 1, the FT-IR spectrum of Cu-MOF shows clearly the removing DMF from Cu-MOF by washing with dichloromethane. Comparison of the diagrams (A) and (B) denotes the deletion of the peak associated with the DMF methyl groups in region 2936. Remove peak in region 3486, indicates a withdrawal of water and carboxylic acid present in the cavity, after the activation of the catalyst.

**Scheme 2:**
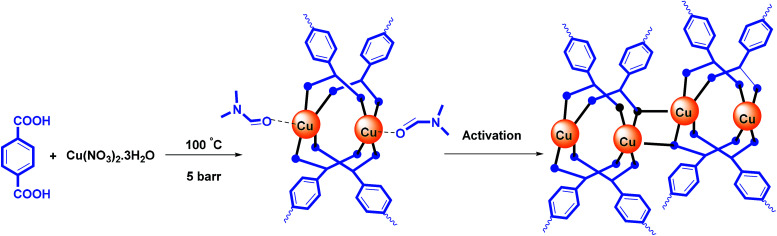
Synthesis and activation of Cu-MOF.

According to the reported literature,^26^ the Cu-MOF structure releases DMF molecules during the activation process, and has active copper sites (Scheme 2).

**Fig. 2:**
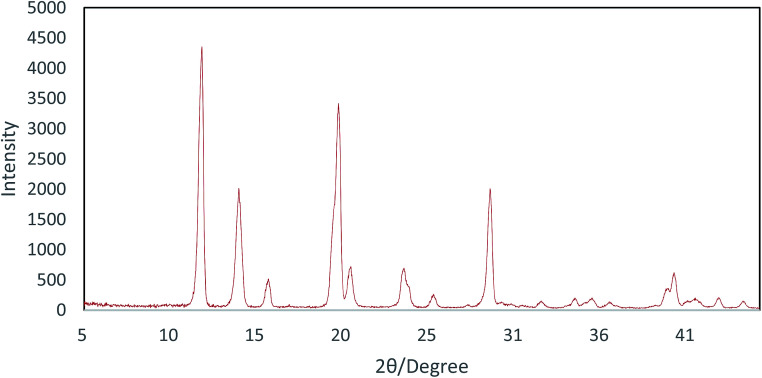
pXRD pattern of Cu-MOF.

The powder XRD pattern of the Cu-MOF compound clearly indicates the presence of copper in its structure and the sharp peak appearing in less than 15 indicates a highly crystalline structure (Fig. 2). According to the XRD spectra, the average size of the crystals (*D*) was 52 nm using the Scherrer equation (1) by replacing *λ* as the wavelength of the X-ray beam, *β*_1/2_ as the line broadening at half the maximum intensity and *θ* as the Bragg's angle.1
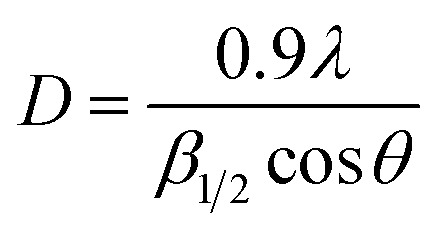


**Fig. 3:**
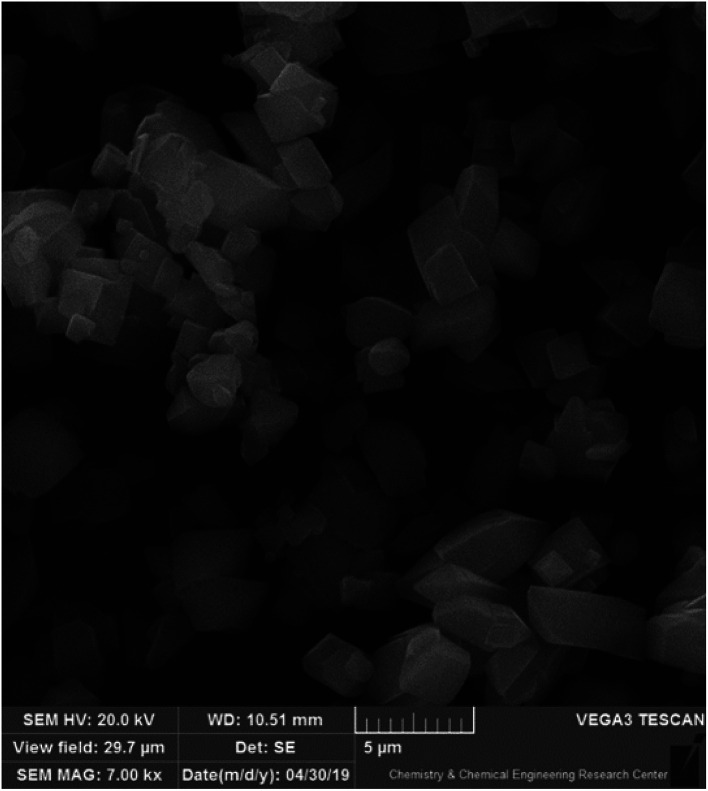
SEM image of cubic structure of Cu-MOF.

SEM techniques can be used to obtain the surface, shape, and appearance of particles. As it clear in Fig. 3, the cubic Cu-MOF structure that was synthesis under pressure is well-defined and it has good agreement with other published articles.^27^

### 3.2. Catalytic behaviors of nano-Cu-MOF for the synthesis tacrine derivatives

The multicomponent reaction of benzaldehyde, malononitrile, 2-hydroxy-1,4-naphthoquinone, and cyclohexanone under different conditions were investigated to determine the optimal conditions for tacrine synthesis. Various solvents such as ethanol, H_2_O, dichloromethane, tetrachloroethylene (C_2_Cl_4_) and 1,2-dichloroethane (EtCl_2_) were screened in the presence of the various catalyst under an air atmosphere. As mentioned before the most of the reported papers on synthesis of tacrine derivatives from the pyranic intermediates, include two or more stages which cause unfavorable higher reaction time and the undesirable less efficiency,^28^ it is also clearly visible in Table 1 (Entry 1 and 2).

**Table 1:** One-pot domino reaction between 2-hydroxy-1,4-naphthoquinone (1 mmol), benzaldehyde (1 mmol), malononitrile (1 mmol) and cyclohexanone (1 mmol) under various conditions

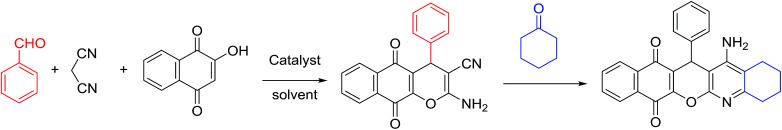				
Entry	Solvent (conditions)^a^	Catalyst	Time (h)	Yield^b^ (%)
1	EtOH (reflux)	NEt_3_ (5 mol%)/AlCl_3_ (2 step)	12	67
2	H_2_O (reflux)	NEt_3_ (5 mol%) AlCl_3_ (2 step)	24	Trace
3	EtOH (reflux)	NEt_3_ (5 mol%) AlCl_3_	24	—
4	EtOH (reflux)	Ni-MOF (3 mg)/AlCl_3_	12	30
5	EtOH (reflux)	Al-MOF	12	20
6	EtOH (reflux)	Cu-MOF (3 mg)/AlCl_3_	24	50
7	EtCl_2_ (reflux)	Cu-MOF (3 mg)/AlCl_3_	5	88
8	CH_2_Cl_2_ (reflux)	Cu-MOF (3 mg)/AlCl_3_	5	40
9	C_2_Cl_4_ (reflux)	Cu-MOF (3 mg)/AlCl_3_	24	—
10	CH_2_Cl_2_ (US)^c^	Cu-MOF (3 mg)/AlCl_3_	12	Trace
11	EtCl_2_ (reflux)	Cu-MOF (3 mg)/AlCl_3_	30	30
12	−(120 °C)	Cu-MOF (3 mg)/AlCl_3_	20	Trace
13	EtOH (reflux)	CuAl-MOF	12	40
14	EtCl_2_ (reflux)	Cu-MOF (4 mg)/AlCl_3_	5	93
15	EtCl_2_ (reflux)	Cu-MOF (5 mg)/AlCl_3_	5	93
16	EtCl_2_ (reflux)	Cu-MOF (2 mg)/AlCl_3_	5	73

a Reaction conditions: benzaldehyde (1 mmol), malononitrile (1 mmol), 2-hydroxy-1,4-naphthoquinone (1 mmol) and cyclohexanone (1.2 mmol).

b Isolated yields.

c Ultrasound at 80% amplitude and was sonicated for 10 min.

In order to optimize the reaction conditions and increase the yield, one step reaction was investigated by using triethylamine and aluminum chloride which does not go further because of undesired interaction between reagents (Entry 3). To overcome this problem, we have used a heterogeneous catalyst, MOF nano catalyst, in dichloroethane as solvent. Our research has revealed that 4 mg catalyst per mmol of reagent has the best efficiency. The optimum condition was achieved by the domino reaction benzaldehyde, malononitrile, 2-hydroxy-1,4-naphthoquinone and cyclohexanone in presence of 4 mg per mmol of Cu-MOF and 1.2 mmol of aluminum chloride.

The elemental analyses, ^1^H, ^13^C NMR, FT-IR spectra and MS of the product clearly indicated the formation of 6a. The IR spectra of 6a exhibited *ν*_max_ at 1631 cm^−1^ for the carbonyl function and 3372 cm ^−1^ and 3452 cm^−1^ for NH_2_. The ^1^H NMR spectra of 6a showed the proton attached to spiro carbon and proton attached to the nitrogen were resonated as a singlet at *δ* 5.26 and 4.20 respectively. The 1H-decoupled ^13^C NMR spectrum of 6a showed 26 distinct resonances in agreement with the proposed structure 6a, carbonyl groups carbon displayed ^13^C resonance signal at *δ* 182.2 and 176.7 ppm, respectively.

After the optimization of reaction conditions, it was generalized by different types of aromatic aldehydes and cycloketones. The domino one-pot synthesis of tacrine derivatives was achieved from the reaction of various aromatic aldehydes, malononitrile, and 2-hydroxy-1,4-naphthoquinone in the presence of Cu-MOF nano catalyst followed by the increase of various ketones and aluminum chloride in short reaction time with high yield as shown in Table 2.

**Table 2:** One-pot, four-component synthesis of new tacrine derivatives in the presence of Cu-MOF and AlCl_3_ in 1,2-dichloroethane under reflux conditions

Entry	Aldehyde	Cycloketone	Yield (%)	Time (h)	Product	Mp (°C)
1	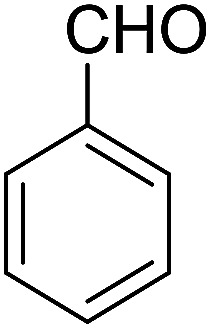	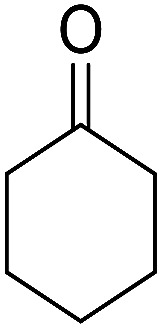	95	5	6a	Dec: 290–293
2	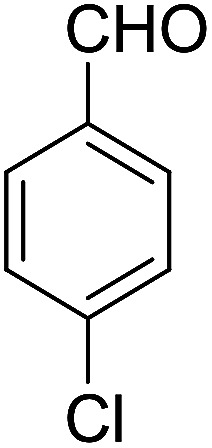	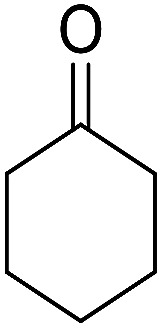	91	3.25	6b	Dec: 290–291
3	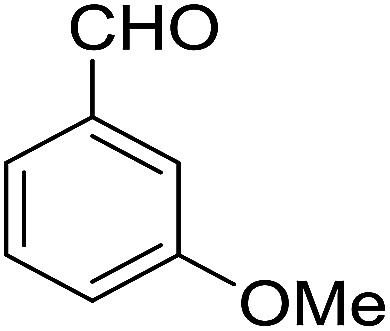	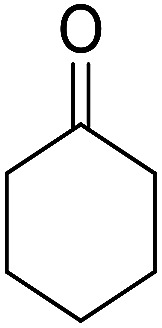	89	3	6c	Dec: 270–271
4	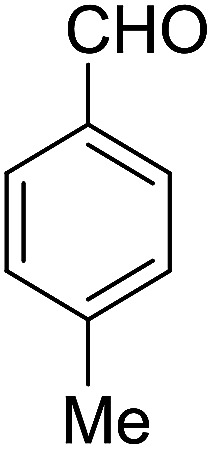	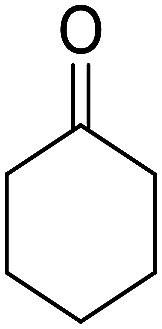	90	3.35	6d	Dec: 242–245
5	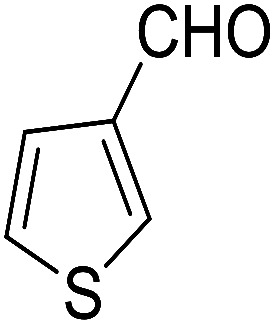	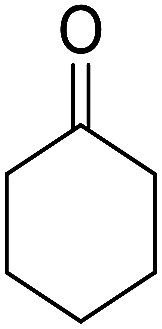	55	6	6e	Dec: 269–271
6	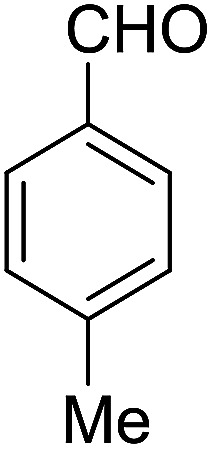	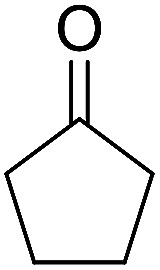	92	2.5	6f	Dec: 300–302
7	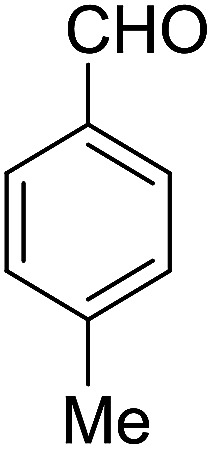	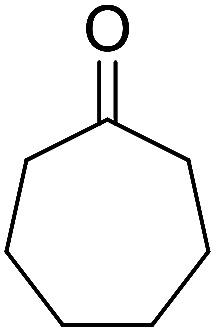	50	4.5	6g	Dec: 263–266
8	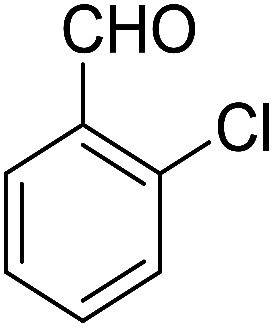	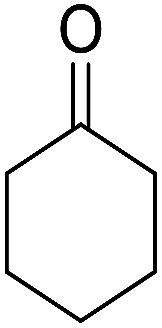	70	3.5	6h	Dec: 274–277
9	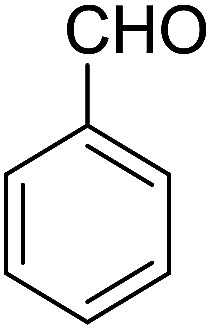	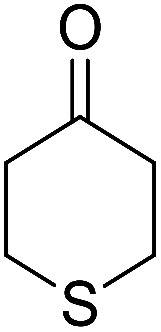	82	4	6i	Dec: 270–273
10	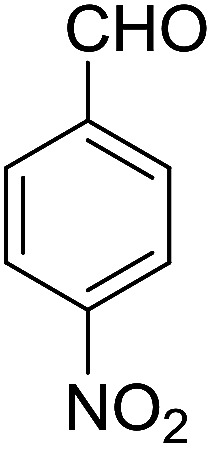	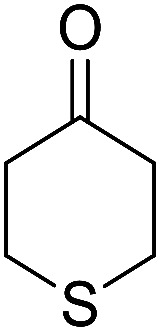	78	5	6j	Dec: 284–286
11	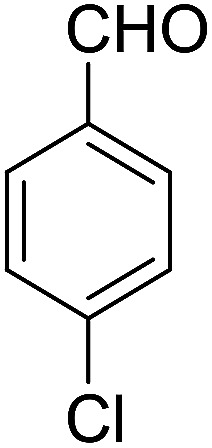	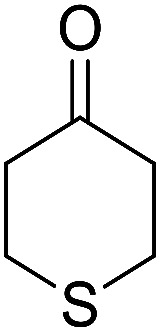	90	4.5	6k	Dec: 250–253

One advantage of using heterogeneous catalysts compared to homogeneous catalysts is their ability to be recycled and reused in chemical reactions, which has attracted particular attention.

### 3.3. Reusability of nano catalyst

**Fig. 4:**
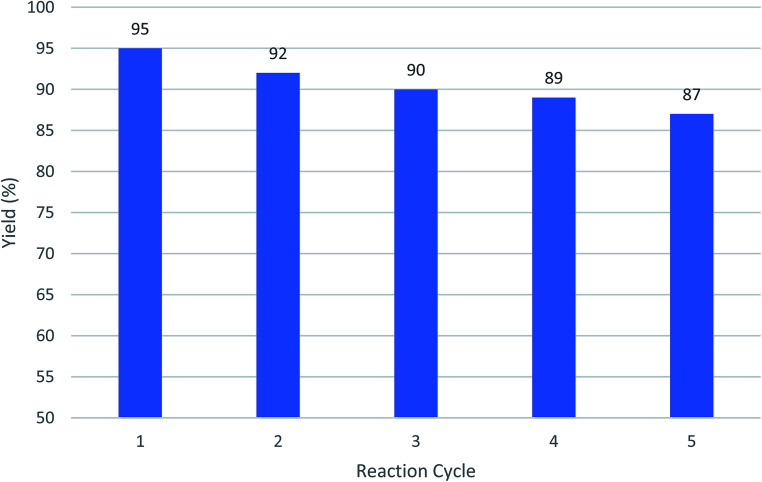
The recycling of Cu-MOF as catalysts using a model reaction of 2-hydroxynaphthalene-1,4-dione, benzaldehyde, malononitrile following by adding cyclohexanone and AlCl_3_.

The ability to the recovery of Cu-MOF catalyst was measured by using after 5 times and no significant change in its catalytic properties was observed. As shown in Fig. 4, the efficiency of the fresh catalyst and the catalyst used have no main different.

Also, FT-IR spectra of the fresh and recycle Cu-MOF catalyst are in good agreement with each other (Fig. S2†).

No leached copper was found in the solution. However, the effect of the homogeneous catalyst was investigated by species leached from the catalyst after hot filtration, and as shown in Fig. S1,† it presented negligible activity in the filtered solution.

#### Representative spectral data

##### 14-Amino-13-phenyl-2,3,4,13-tetrahydro-1*H*-benzo[6,7]chromeno[2,3-*b*]quinoline-7,12-dione (6a)

Yield 93 (%); yellow solid; mp = 290–293 dec °C; IR (KBr): 3464, 3376 (NH_2_), 1631, 1595 (C=O) cm^−1^. ^1^H NMR (500 MHz, CDCl_3_) *δ* = 8.44–7.83 (m, 2H), 7.78–7.57 (m, 2H), 7.54–7.15 (m, 5H), 5.24 (s, 1H), 4.19 (s, 2H, NH_2_), 2.87–2.80 (m, 2H), 2.45–2.23 (m, 2H), 1.92–1.77 (m, 4H) ppm; ^13^C NMR (125 MHz, CDCl_3_) *δ* = 182.2, 176.7, 154.0, 152.8, 149.7, 149.2, 140.6, 133.0, 132.4, 130.7, 129.8 (2C), 127.9 (2C), 126.6, 125.4, 125.2, 121.5, 113.6, 97.6, 54.2, 34.9, 31.4, 21.9, 21.5, 21.2 ppm; MS (70 eV) *m*/*z* 408 [M^+^], 331, 105, 77; anal. calcd for C_26_H_20_N_2_O_3_: C, 76.46; H, 4.94; N, 6.86. Found: C, 76.50; H, 4.83; N, 6.91.

##### 14-Amino-13-(4-chlorophenyl)-2,3,4,13-tetrahydro-1*H*-benzo[6,7]chromeno[2,3-*b*]quinoline-7,12-dione (6b)

Yield 91 (%); yellow solid; mp = 290–291 dec °C; IR (KBr): 3463, and 3376 (NH_2_), 1631, 1595 (C=O) cm^−1^; ^1^H NMR (500 MHz, CDCl_3_) *δ* = 8.15 (dd, *J* = 4.0, 5.0 Hz, 1H), 7.98 (dd, *J* = 4.0, 5.0 Hz, 1H), 7.70 (d, *J* = 5.0 Hz, 1H), 7.69 (d, *J* = 5.0 Hz, 1H), 7.38 (d, *J* = 8.5 Hz, 2H), 7.23 (d, *J* = 8.5 Hz, 2H), 5.27 (s, 1H), 4.31 (s, 2H, NH_2_), 2.82–2.81 (m, 2H), 2.42–2.38 (m, 2H), 1.88–1.81 (m, 4H) ppm; ^13^C NMR (125 MHz, CDCl_3_) *δ* = 182.3, 176.8, 154.2, 152.7, 149.8, 149.4, 139.2, 133.2, 132.6, 132.5, 130.6, 129.8, 129.2, 128.1, 125.6, 125.3, 121.2, 113.8, 97.0, 34.3, 31.5, 22.0, 21.5, 21.2 ppm; MS (70 eV) *m*/*z* 442 [M^+^], 331, 105, 77; anal. calcd for C_26_H_19_ClN_2_O_3_: C, 70.51; H, 4.32; N, 6.33. Found: 70.43; H, 4.45; N, 6.38.

##### 14-Amino-13-(3-methoxyphenyl)-2,3,4,13-tetrahydro-1*H*-benzo[6,7]chromeno[2,3-*b*]quinoline-7,12-dione (6c)

Yield 89 (%); yellow solid; mp = 270–271 dec °C; IR (KBr): 3472, 3375 (NH_2_),1682, 1634, 1594 cm^−1^. ^1^H NMR (500 MHz, CDCl_3_) *δ* = 8.17 (dd, *J* = 5.8, 3.3 Hz, 1H), 8.03 (dt, *J* = 7.3, 3.8 Hz, 1H), 7.70 (dt, *J* = 7.1, 3.6 Hz, 2H), 7.23 (t, *J* = 7.9 Hz, 2H), 7.07 (d, *J* = 7.6 Hz, 1H), 6.99 (t, *J* = 2.2 Hz, 1H), 6.76 (dd, *J* = 8.3, 2.6 Hz, 1H), 5.23 (s, 1H), 4.21 (s, 2H, NH_2_), 3.76 (s, 3H, OMe), 2.84 (s, 2H), 2.49–2.20 (m, 2H), 1.86 (s, 4H); ^13^C NMR (125 MHz, CDCl_3_) *δ* = 182.2, 161.2, 159.0, 154.2, 152.6, 149.8, 142.3, 133.0, 132.5, 130.7, 129.9, 128.9, 125.5, 125.3 (2C), 120.3 (2C), 114.0, 113.9, 113.66, 111.7, 54.2, 35.0, 31.4, 21.9, 21.4, 21.2 ppm; MS (70 eV) *m*/*z* 438 [M^+^], 331, 92, 77; anal. calcd for C_27_H_22_N_2_O_4_: C, 73.96; H, 5.06; N, 6.39. Found: C, 73.89; H, 5.15; N, 6.33.

##### 14-Amino-13-(*p*-tolyl)-2,3,4,13-tetrahydro-1*H*-benzo[6,7]chromeno[2,3-*b*]quinoline-7,12-dione (6d)

Yield 90 (%); yellow solid; mp = 242–245 dec °C; IR (KBr) = 3472, 3375 (NH_2_), 1734, 1635, 1594 cm^−1^. ^1^H NMR (500 MHz, CDCl_3_) *δ* = 8.39–7.94 (m, 2H), 7.81–7.54 (m, 2H), 7.41–7.30 (m, 2H), 7.11 (d, *J* = 7.9 Hz, 2H), 5.25 (s, 1H), 4.20 (s, 2H, NH_2_), 2.97 (h, *J* = 9.2 Hz, 2H), 2.79–2.50 (m, 2H), 2.28 (s, 3H, Me), 2.21–2.09 (m, 2H), 2.07 (s, 1H), 1.28 (t, *J* = 7.2 Hz, 1H) ppm; ^13^C NMR (125 MHz, CDCl_3_) *δ* = 182.4, 177.0, 162.0, 155.0, 149.2, 147.8, 137.9, 136.5, 133.0, 132.5, 130.7, 129.8, 128.6 (2C), 127.7 (2C), 125.5, 125.3, 121.6, 118.3, 98.2, 33.3, 33.2, 26.0, 21.2, 20.0, 13.1 ppm; MS (70 eV) *m*/*z* 442 [M^+^], 331, 105, 91; anal. calcd for C_27_H_22_N_2_O_3_: C, 76.76; H, 5.25; N, 6.63. Found: C, 76.70; H, 5.19; N, 6.70.

##### 14-Amino-13-(thiophen-2-yl)-2,3,4,13-tetrahydro-1*H*-benzo[6,7]chromeno[2,3-*b*]quinoline-7,12-dione (6e)

Yield 55 (%); brown solid; mp = 269–271 dec °C; IR (KBr) = 3472, 3378 (NH_2_), 1734, 1678, 1635, 1594 cm^−1^. ^1^H NMR (500 MHz, DMSO-*d*_6_) *δ* = 8.11–8.04 (m, 1H), 8.03–7.97 (m, 1H), 7.92–7.66 (m, 2H), 7.27 (d, *J* = 5 Hz, 1H), 7.0 (d, *J* = 3.5 Hz, 1H), 6.86 (dd, *J* = 5.1, 3.6 Hz, 1H), 6.03 (s, 2H, NH_2_), 5.84 (s, 1H), 2.61 (d, *J* = 5.5 Hz, 2H), 2.46–2.13 (m, 2H), 1.34–0.97 (m, 2H) ppm; ^13^C NMR (125 MHz, DMSO-*d*_6_) *δ* = 182.0, 177.3, 152.7, 152.4, 151.2, 149.7, 145.5, 134.0, 133.4, 130.6, 130.0, 126.0, 125.5, 125.5, 125.3, 124.5, 121.8, 113.2, 96.7, 31.4, 28.4, 22.5, 21.7, 21.4 ppm; MS (70 eV) *m*/*z* 414 [M^+^], 331, 105, 77; anal. calcd for C_24_H_18_N_2_O_3_S: C, 69.55; H, 4.38; N, 6.76; S, 7.74. Found: C, 69.50; H, 4.32; N, 6.84; S, 7.79.

##### 13-Amino-12-(*p*-tolyl)-1,2,3,12-tetrahydrobenzo[6,7]chromeno[2,3-*b*]cyclopenta[*e*]pyridine-6,11-dione (6f)

Yield 92 (%); yellow solid; mp = 300–302 dec °C; IR (KBr) = 3445, 3355 (NH_2_), 1731, 1644, 1582 cm^−1^. ^1^H NMR (500 MHz, CDCl_3_) *δ* = 8.27–7.92 (m, 2H), 7.76–7.59 (m, 2H), 7.34 (d, *J* = 8.0 Hz, 2H), 7.11 (d, *J* = 7.8 Hz, 2H), 5.25 (s, 1H), 4.18 (s, 2H, NH_2_), 2.96 (hept, *J* = 8.2, 7.3 Hz, 2H), 2.81–2.48 (m, 2H), 2.28 (s, 3H), 2.07 (s, 1H), 1.28 (t, *J* = 7.1 Hz, 1H) ppm; ^13^C NMR (125 MHz, CDCl_3_) *δ* = 182.4, 162.0, 155.0, 149.1, 147.8, 137.9, 136.4, 133.0, 132.5, 130.7, 129.8, 128.7, 127.7, 125.5, 125.2, 121.5, 118.2, 98.1, 34.5, 33.2, 26.1, 21.2, 20.0, 13.1 ppm; MS (70 eV) *m*/*z* 408 [M^+^], 317, 91, 77; anal. calcd for C_26_H_20_N_2_O_3_: C, 76.46; H, 4.94; N, 6.86. Found: C, 76.50; H, 4.82; N, 6.92.

##### 13-Amino-14-(*p*-tolyl)-8,9,10,11,12,14-hexahydrobenzo[6,7]chromeno[2,3-*b*]cyclohepta[*e*]pyridine-5,15-dione (6g)

Yield 50 (%); brownish solid; mp = 263–266 dec °C; IR (KBr) = 3465, 3378 (NH_2_), 1641, 1599 cm^−1^. ^1^H NMR (500 MHz, CDCl_3_) *δ* = 8.14 (s, 1H), 7.99 (s, 1H), 7.67 (s, 2H), 7.33 (d, *J* = 7.9 Hz, 2H), 7.0 (d, *J* = 8.5 Hz, 2H), 5.21 (s, 1H), 4.25 (s, 2H, NH_2_), 2.81 (s, 2H), 2.30–2.12 (m, 4H), 1.9–1.4 (m, 5H), 1.26 (d, *J* = 7.8 Hz, 2H) ppm; ^13^C NMR (125 MHz, CDCl_3_) *δ* = 182.4, 176.8, 161.6, 158.7, 153.8, 149.8, 137.8, 136.4, 133.0, 132.4, 130.7, 129.8, 128.6 (2C), 127.7 (2C), 125.5, 125.2, 121.6, 113.6, 97.7, 34.5, 31.4, 21.9, 21.5, 21.2, 20.0, 13.1 ppm; MS (70 eV) *m*/*z* 436 [M^+^], 421, 345, 91; anal. calcd for C_28_H_24_N_2_O_3_: C, 77.04; H, 5.54; N, 6.42. Found: C, 77.02; H, 5.59; N, 6.49.

##### 14-Amino-13-(2-chlorophenyl)-2,3,4,13-tetrahydro-1*H*-benzo[6,7]chromeno[2,3-*b*]quinoline-7,12-dione (6h)

Yield 70 (%); yellow solid; mp = 274–277 dec °C; IR (KBr) = 3451, 3383 (NH_2_), 1636, 1597 cm^−1^. ^1^H NMR (500 MHz, CDCl_3_) *δ* = 8.26–8.09 (m, 1H), 8.09–7.92 (m, 1H), 7.79–7.61 (m, 2H), 7.44–7.32 (m, 1H), 7.15 (tt, *J* = 7.4, 5.4 Hz, 2H), 5.63 (s, 1H), 4.54 (s, 2H, NH_2_), 2.96–2.61 (m, 2H), 2.45–2.14 (m, 2H), 1.88–1.65 (m, 4H) ppm; ^13^C NMR (125 MHz, CDCl_3_) *δ* = 182.1, 176.8, 153.8, 152.4, 150.1, 149.8, 138.9, 133.2, 132.5, 130.4, 130.5, 130.2, 129.8, 128.5, 127.8, 127.0, 125.6, 125.3, 121.4, 113.3, 97.1, 31.7, 31.3, 22.0, 21.4, 21.2 ppm; MS (70 eV) *m*/*z* 442 [M^+^], 407, 331, 105; anal. calcd for C_26_H_19_ClN_2_O_3_: C, 70.51; H, 4.32; N, 6.33. Found: C, 70.48; H, 4.31; N, 6.40.

##### 14-Amino-13-phenyl-4,13-dihydro-1*H*,3*H*-benzo[6,7]chromeno[2,3-*b*]thiopyrano[3,4-*e*]pyridine-7,12-dione (6i)

Yield 82 (%); yellow solid; mp = 270–273 dec °C; IR (KBr) = 3451, 3376 (NH_2_), 1657, 1595 cm^−1^. ^1^H NMR (500 MHz, DMSO-*d*_6_) *δ* = 8.07–8.00 (m, 1H), 7.93 (d, *J* = 4.8 Hz, 1H), 7.85–7.77 (m, 2H), 7.57–7.46 (m, 2H), 7.30–6.9 (m, 5H), 5.54 (s, 1H), 3.64–3.53 (m, 1H), 3.40 (d, *J* = 16.2 Hz, 1H), 3.00 (q, *J* = 6.2 Hz, 2H), 2.88 (d, *J* = 6.1 Hz, 2H) ppm; ^13^C NMR (125 MHz, DMSO-*d*_6_) *δ* = 181.7, 176.1, 152.9, 150.7, 148.6, 148.3, 148.3, 140.8, 134.1, 133.7, 130.3, 129.9, 128.2 (2C), 128.1, 127.8 (2C), 126.7, 125.5, 125.4, 122.7, 111.8, 32.6, 30.4, 23.0, 22.6 ppm; MS (70 eV) *m*/*z* 426 [M^+^], 349, 315, 303; anal. calcd for C_25_H_18_N_2_O_3_S: C, 70.41; H, 4.25; N, 6.57; S, 7.52. Found: C, 70.40; H, 4.23; N, 6.63; S, 7.61.

##### 14-Amino-13-(4-nitrophenyl)-4,13-dihydro-1*H*,3*H*-benzo[6,7]chromeno[2,3-*b*]thiopyrano[3,4-*e*]pyridine-7,12-dione (6j)

Yield 78 (%); yellow solid; mp = 284–286 dec °C; IR (KBr) = 3490, 3360 (NH_2_), 1637, 1592 cm^−1^. ^1^H NMR (500 MHz, DMSO-*d*_6_) *δ* = 8.10 (d, *J* = 8.3 Hz, 2H), 8.08–8.02 (m, 1H), 7.96–7.90 (m, 1H), 7.86–7.79 (m, 2H), 7.77 (d, *J* = 8.2 Hz, 2H), 6.19 (s, 2H, NH_2_), 5.69 (s, 1H), 3.57 (d, *J* = 16.3 Hz, 1H), 3.38 (d, *J* = 16.0 Hz, 1H), 2.94 (q, *J* = 6.0, 5.4 Hz, 2H), 2.85 (h, *J* = 8.2, 7.3 Hz, 2H) ppm; ^13^C NMR (125 MHz, DMSO-*d*_6_) *δ* = 182.0, 177.0, 152.7, 152.0, 150.7, 150.1, 149.2, 145.7, 133.9, 133.4, 130.5, 130.0, 129.5 (2C), 125.5, 125.2, 122.7 (2C), 121.0, 111.9, 96.8, 33.3, 32.9, 24.0, 22.9 ppm; MS (70 eV) *m*/*z* 471 [M^+^], 349, 315, 303; anal. calcd for C_25_H_17_N_3_O_5_S: C, 63.69; H, 3.63; N, 8.91; S, 6.80. Found: C, 63.60; H, 3.68; N, 8.88; S, 6.76.

##### 14-Amino-13-(4-chlorophenyl)-4,13-dihydro-1*H*,3*H*-benzo[6,7]chromeno[2,3-*b*]thiopyrano[3,4-*e*]pyridine-7,12-dione (6k)

Yield 90 (%); yellow solid; mp = 250–253 dec °C; IR (KBr) = 3450, 3345 (NH_2_), 1665, 1595 cm^−1^. ^1^H NMR (500 MHz, DMSO-*d*_6_) *δ* = 8.13–8.01 (m, 1H), 8.00–7.91 (m, 1H), 7.85 (t, *J* = 4.6 Hz, 2H), 7.55 (d, *J* = 8.1 Hz, 2H), 7.32 (d, *J* = 8.1 Hz, 2H), 7.09 (s, 2H, NH_2_), 5.59 (s, 1H), 3.60 (d, *J* = 16.3 Hz, 1H), 3.40 (d, *J* = 16.2 Hz, 1H), 3.08–2.81 (m, 4H) ppm; ^13^C NMR (125 MHz, CDCl_3_) *δ* = 182.8, 176.5, 152.9, 150.7, 148.8, 139.7, 134.1, 133.6, 131.4, 130.5, 130.2 (2C), 130.0, 127.7 (2C), 125.5, 125.4, 122.0, 112.0, 97.5, 32.1, 30.5, 23.1, 22.6 ppm; MS (70 eV) *m*/*z* 460 [M^+^], 349, 315, 303; anal. calcd for C_25_H_17_ClN_2_O_3_S: C, 65.15; H, 3.72; 6.08; S, 6.96. Found: C, 65.10; H, 3.83; 6.18; S, 6.90.

## 4. Conclusion

In conclusion, we have effectively developed and expanded simple and straightforward procedure for one-pot synthesis of tacrine derivatives. One-pot, domino four-component reaction of aldehydes, malononitrile, 2-hydroxy-1,4-naphthoquinone, and cycloketones was carried out in the presence of Cu-MOF and aluminum chloride. Without the requirement to isolate the pyranic intermediate and in a single container, the reaction was conducted under reflux to give the desired products in high yields. Prominent features of the current procedure include a step-economic increasing yield, economic feasibility, capability of recycling the catalyst and covering a broad substrate scope. The heterogeneous nanocatalyst Cu-MOF presented excellent catalytic performance and can be recycled simply by centrifuging; it can also recover 5 times without considerable loss of efficiency.

We expect this method to be performed easily by pharmacists and chemists and biologically active tacrine derivatives that were synthesized over several stages can be easily synthesized without the need to separate intermediates and purify them.

## Conflicts of interest

There are no conflicts to declare.

## Supplementary Material

RA-010-C9RA10111J-s001
