# Supplementary material for: Cu-metal organic frameworks (Cu-MOF) as an environment-friendly and economical catalyst for one pot synthesis of tacrine derivatives
Source: RSC Adv. 2020 Jan 9;10(4):1995–2003. doi: 10.1039/c9ra10111j (PMC9047972; doi:10.1039/c9ra10111j)

Electronic Supplementary Material (ESI) for RSC Advances.
This journal is © The Royal Society of Chemistry 2020

Cu- Metal organic frameworks (Cu-MOF) as an environment-friendly and economically catalyst for one pot synthesis of tacrine derivatives

Hoda Mollabagher[a], Salman Taheri[a*], Mohammad majid Mojtahedi[a], SeyedAmirhossein Seyedmousavi[b]

[a], Chemistry and Chemical Engineering Research Center of Iran, PO Box 14115-186, Tehran, Iran

[b]Process Engineering Department, Faculty of Chemical Engineering, Tarbiat Modares University, Tehran, Iran

Figure S1. Leaching test shows no contribution of active species as homogeneous catalyst in the reaction medium.

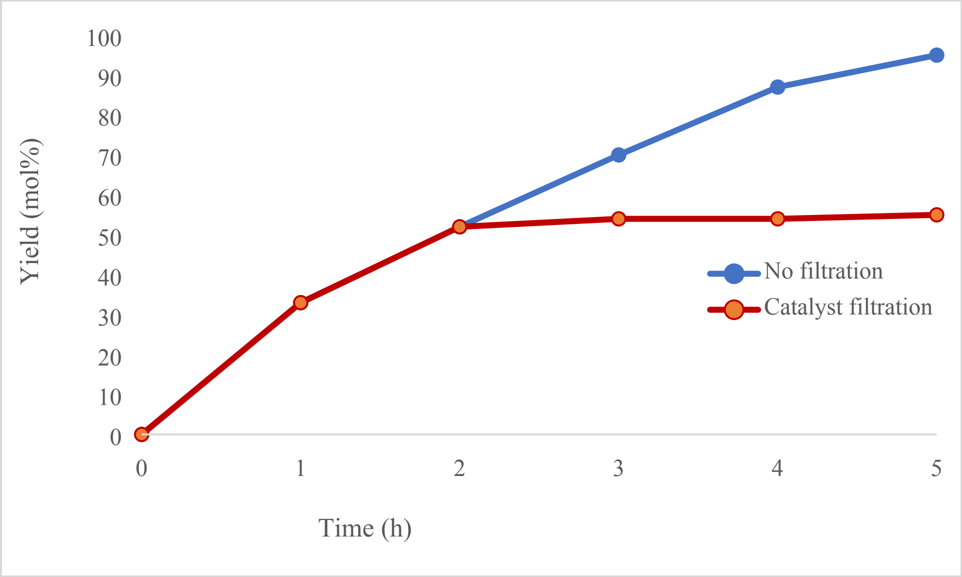

Figure S2. FT-IR spectra of the fresh (a) and recycle (b) Cu-MOF catalyst.

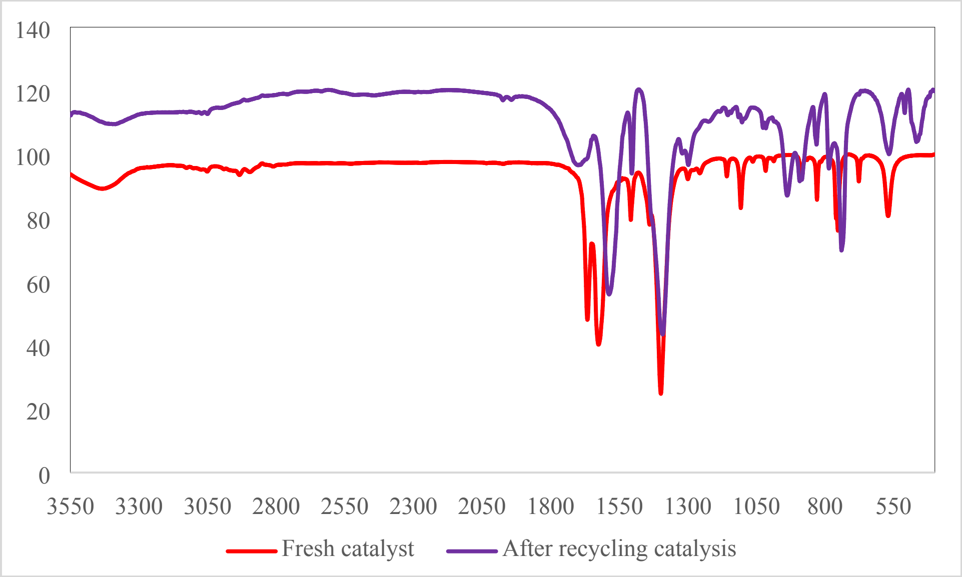

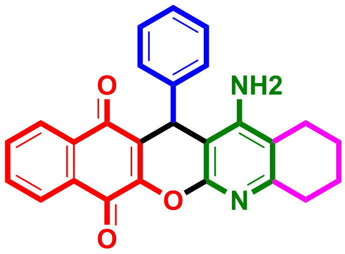

6a

Figure S3. FT-IR spectrum of 14-Amino-13-phenyl-2,3,4,13-tetrahydro-1H-benzo[6,7]chromeno[2,3-b]quinoline-7,12-dione

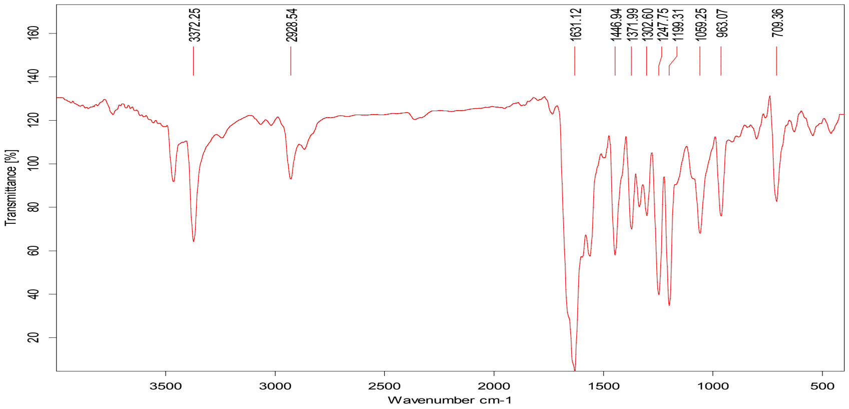

Figure S4. Mass spectrum of 14-Amino-13-phenyl-2,3,4,13-tetrahydro-1H-benzo[6,7]chromeno[2,3-b]quinoline-7,12-dione

File : C:\MSDCHEM\3\DATA\Snapshot\30001203.D
Operator :
Acquired : 5 May 2019 10:24 using AcqMethod PAH
Instrument : Instrumen
Sample Name: BML2
Misc Info :
Vial Number: 1

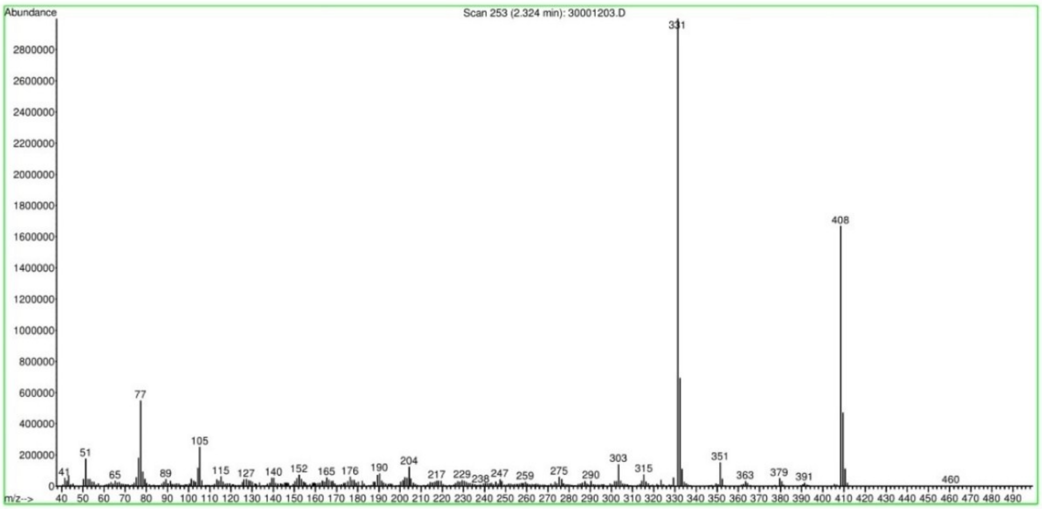

Figure S5. ^{1}**H NMR spectrum of 14-Amino-13-phenyl-2,3,4,13-tetrahydro-1H-benzo[6,7]chromeno[2,3-b]quinoline-7,12-dione**

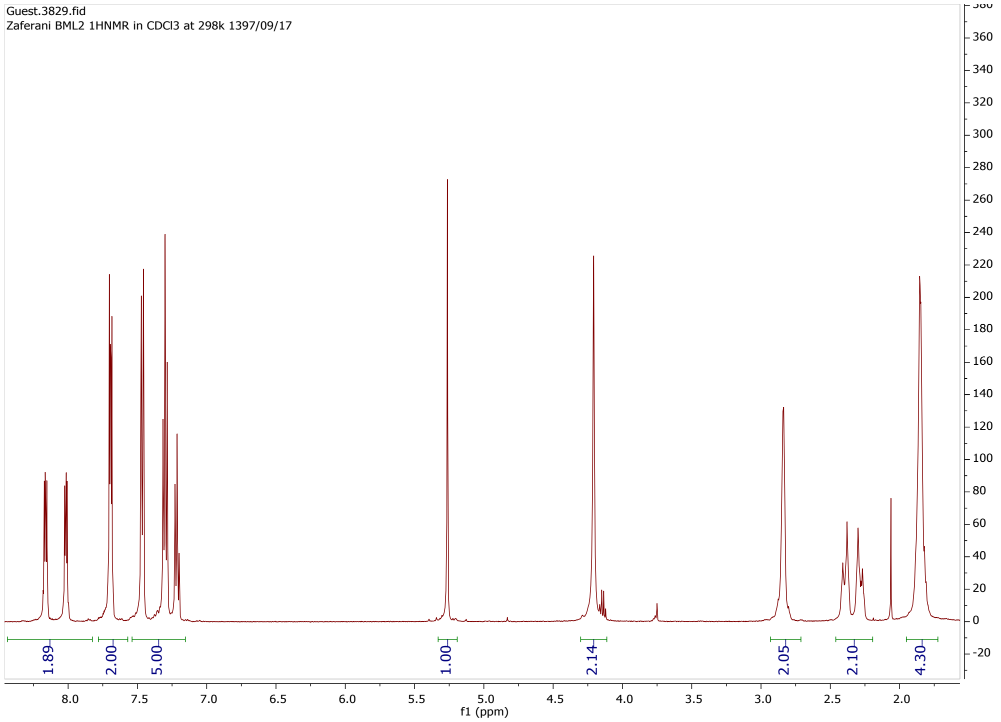

Figure S6. ^{13}**C NMR spectrum of 14-Amino-13-phenyl-2,3,4,13-tetrahydro-1H-benzo[6,7]chromeno[2,3-b]quinoline-7,12-dione**

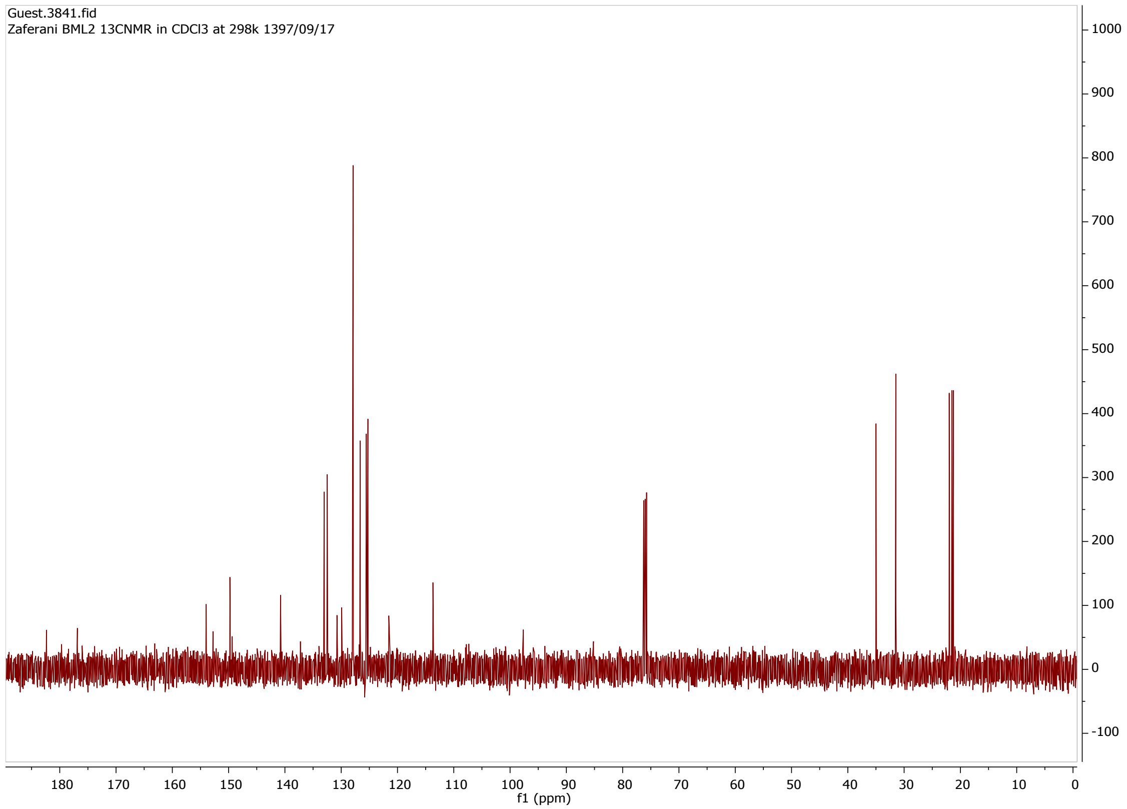

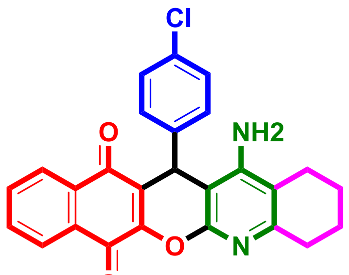

6b

Figure S7. FT-IR spectrum of 14-Amino-13-(4-chlorophenyl)-2,3,4,13-tetrahydro-1H-benzo[6,7]chromeno[2,3-b]quinoline-7,12-dione

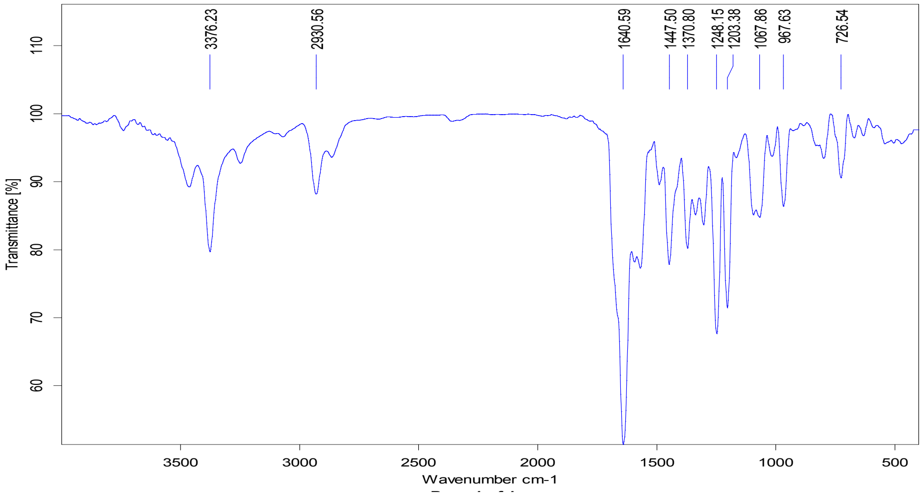

Figure S8. Mass spectrum of 14-Amino-13-(4-chlorophenyl)-2,3,4,13-tetrahydro-1H-benzo[6,7]chromeno[2,3-b]quinoline-7,12-dione

File : C:\MSDCHEM\3\DATA\Snapshot\30001202.D
Operator :
Acquired : 5 May 2019 10:13 using AcqMethod PAH
Instrument : Instrumen
Sample Name: 4ClMl2
Misc Info :
Vial Number: 1

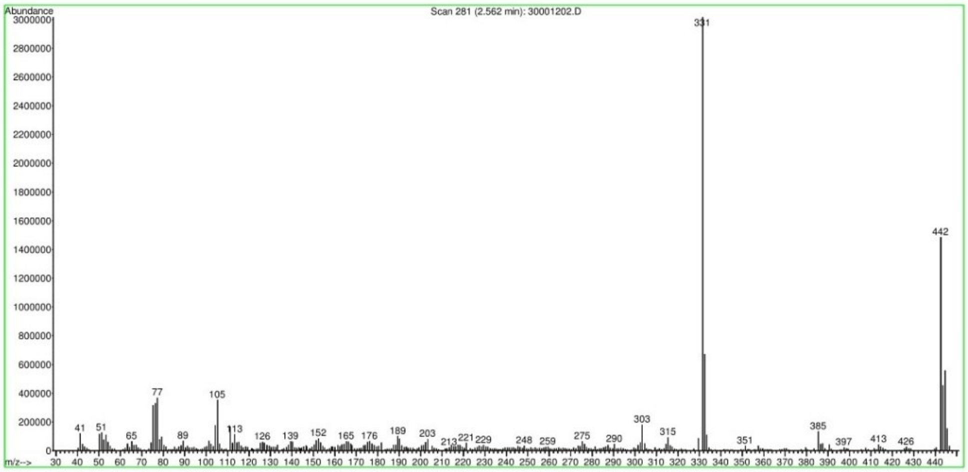

Figure S9. ^{1}H NMR spectrum of 14-Amino-13-(4-chlorophenyl)-2,3,4,13-tetrahydro-1H-benzo[6,7]chromeno[2,3-b]quinoline-7,12-dione

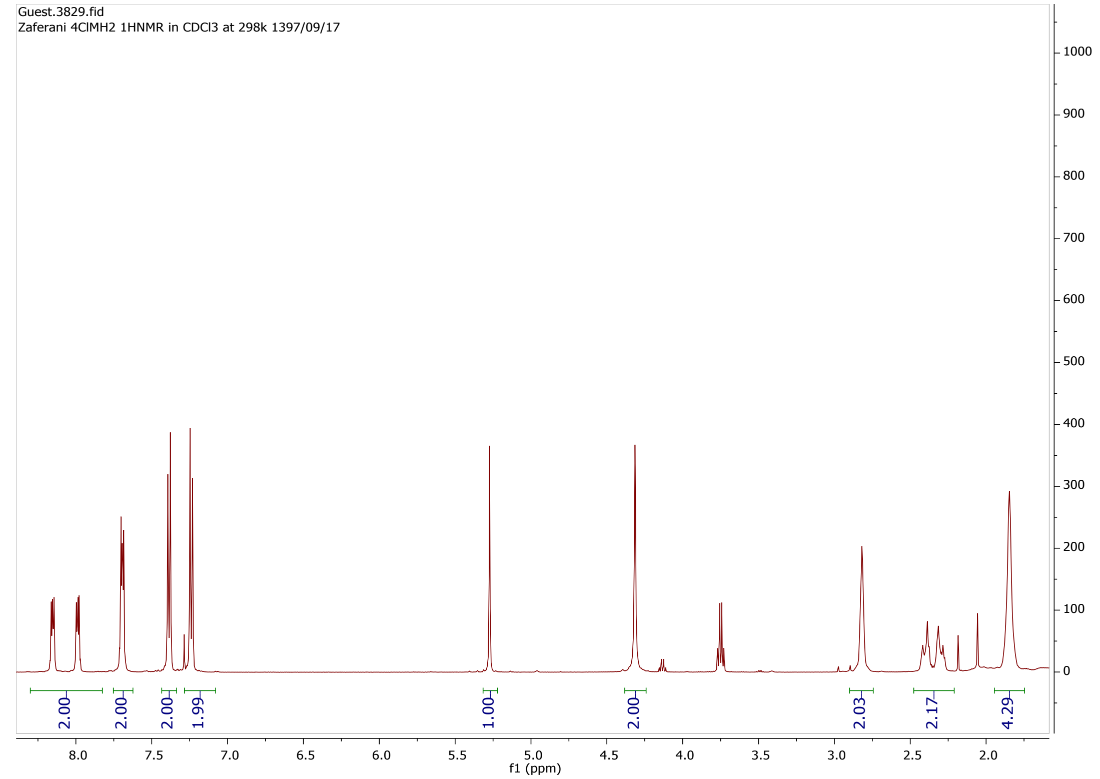

Figure S10. ^{13}C NMR spectrum of 14-Amino-13-(4-chlorophenyl)-2,3,4,13-tetrahydro-1H-benzo[6,7]chromeno[2,3-b]quinoline-7,12-dione

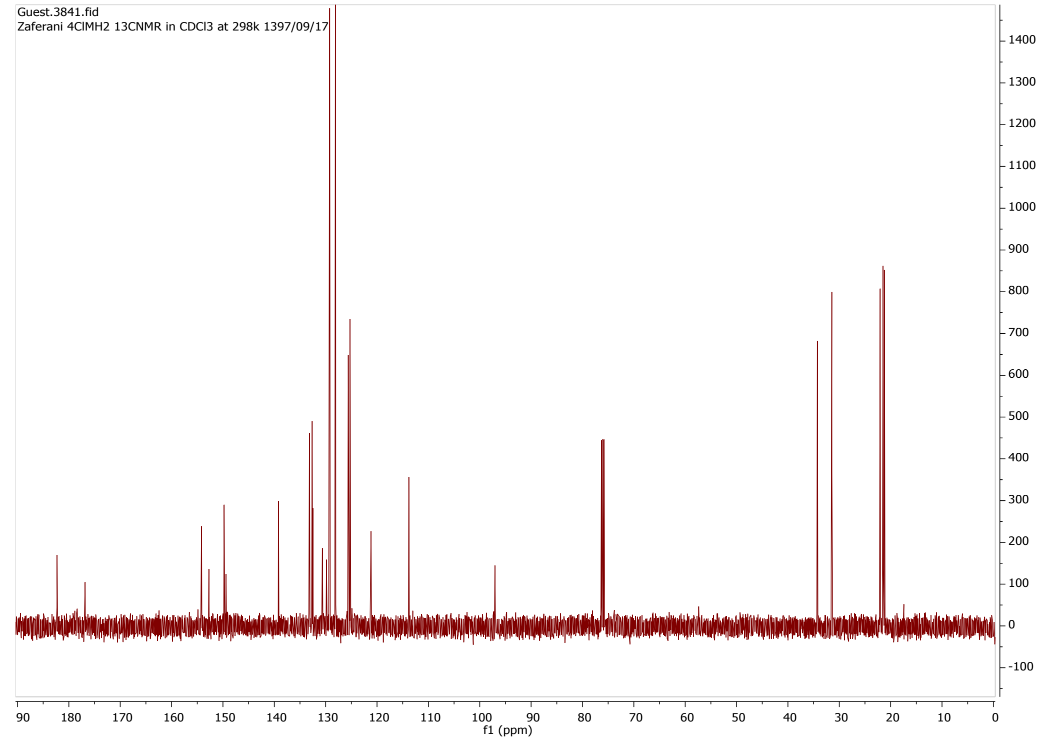

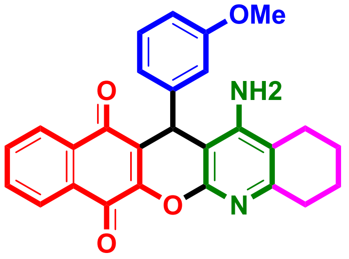

6c

Figure S11. FT-IR spectrum of 14-Amino-13-(3-methoxyphenyl)-2,3,4,13-tetrahydro-1*H*-benzo[6,7]chromeno[2,3-b]quinoline-7,12-dione

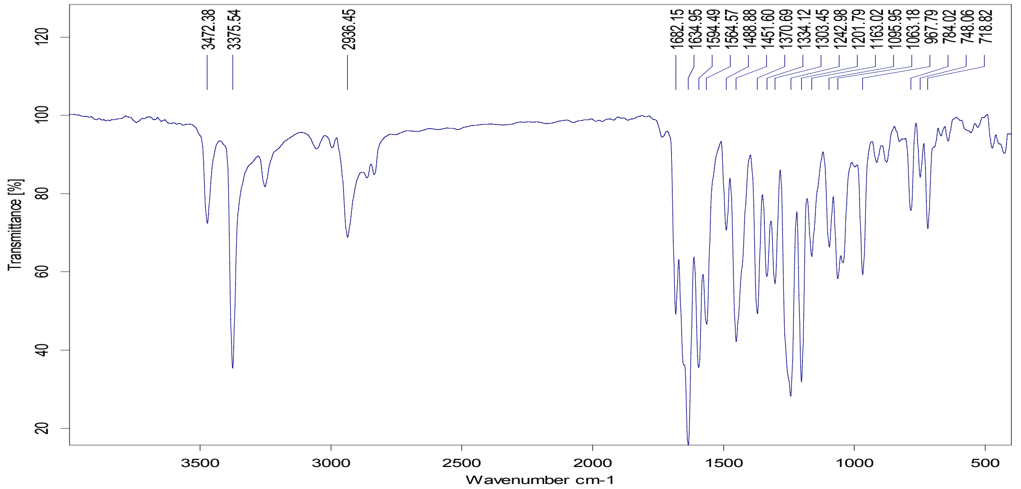

Figure S12. Mass spectrum of 14-Amino-13-(3-methoxyphenyl)-2,3,4,13-tetrahydro-1*H*-benzo[6,7]chromeno[2,3-b]quinoline-7,12-dione

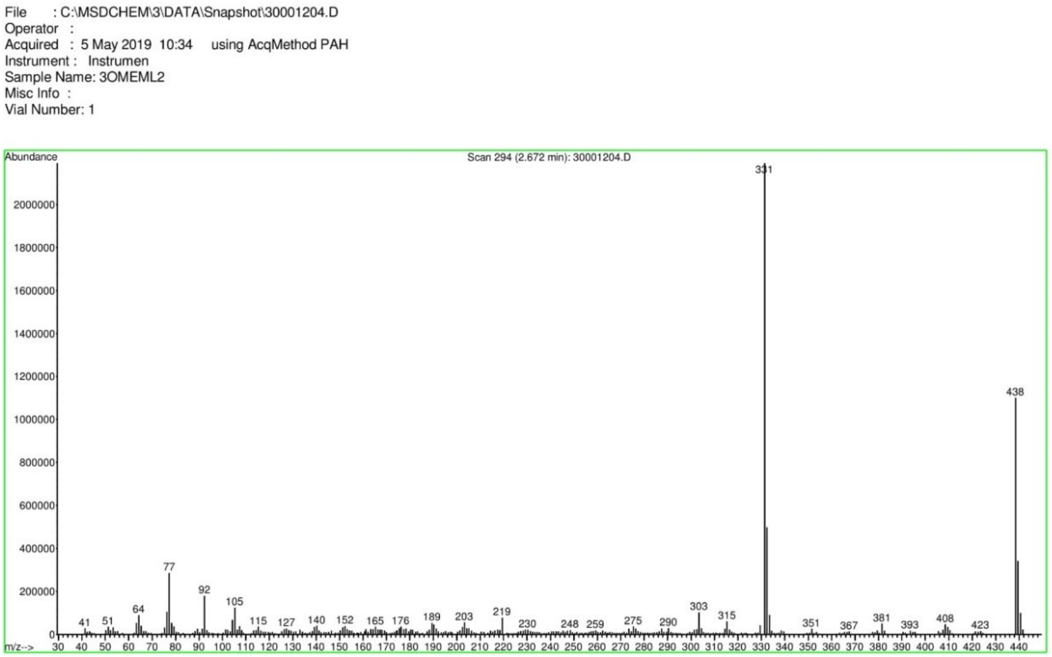

Figure S13. ^{1}**H NMR spectrum of 14-Amino-13-(3-methoxyphenyl)-2,3,4,13-tetrahydro-1*H*-benzo[6,7]chromeno[2,3-b]quinoline-7,12-dione**

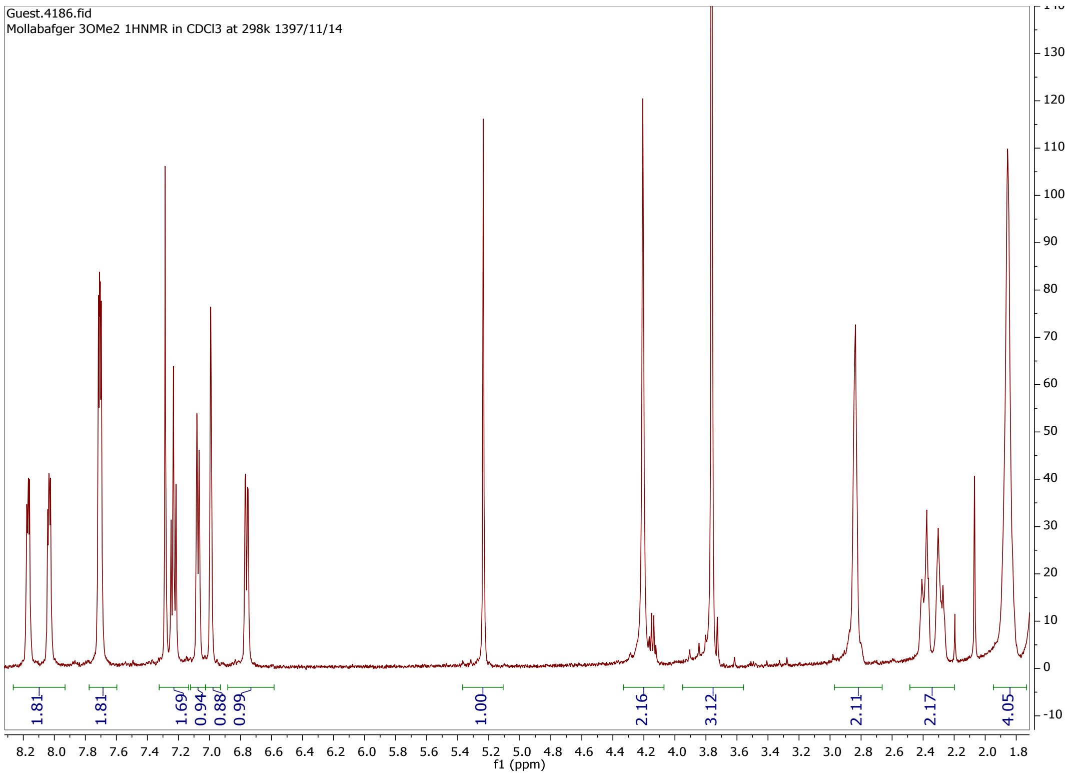

Figure S14. ^{13}**C NMR spectrum of 14-Amino-13-(3-methoxyphenyl)-2,3,4,13-tetrahydro-1*H*-benzo[6,7]chromeno[2,3-b]quinoline-7,12-dione**

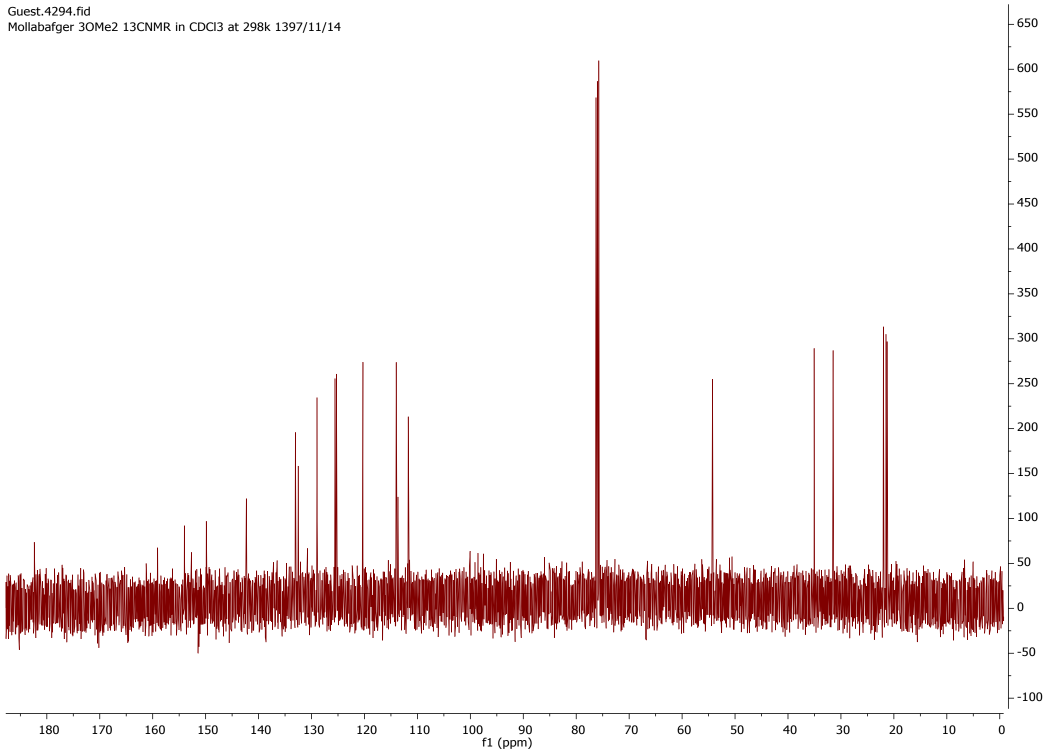

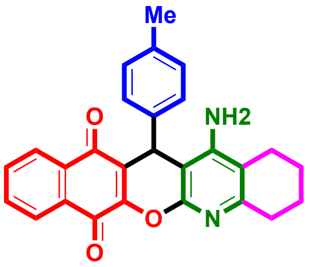

6d

Figure S15. FT-IR spectrum of 14-Amino-13-(p-tolyl)-2,3,4,13-tetrahydro-1*H*-benzo[6,7]chromeno[2,3-b]quinoline-7,12-dione

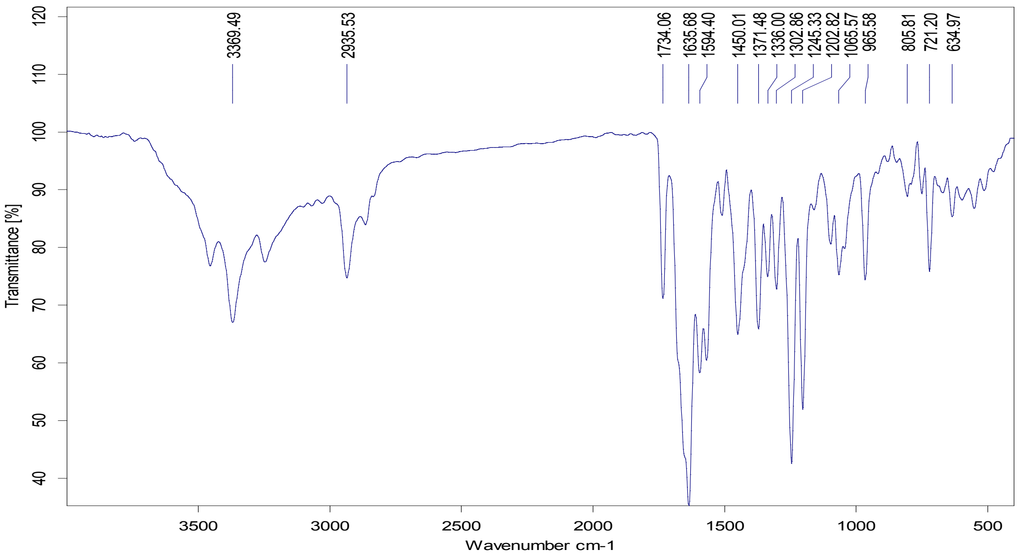

Figure S16. Mass spectrum of 14-Amino-13-(p-tolyl)-2,3,4,13-tetrahydro-1*H*-benzo[6,7]chromeno[2,3-b]quinoline-7,12-dione

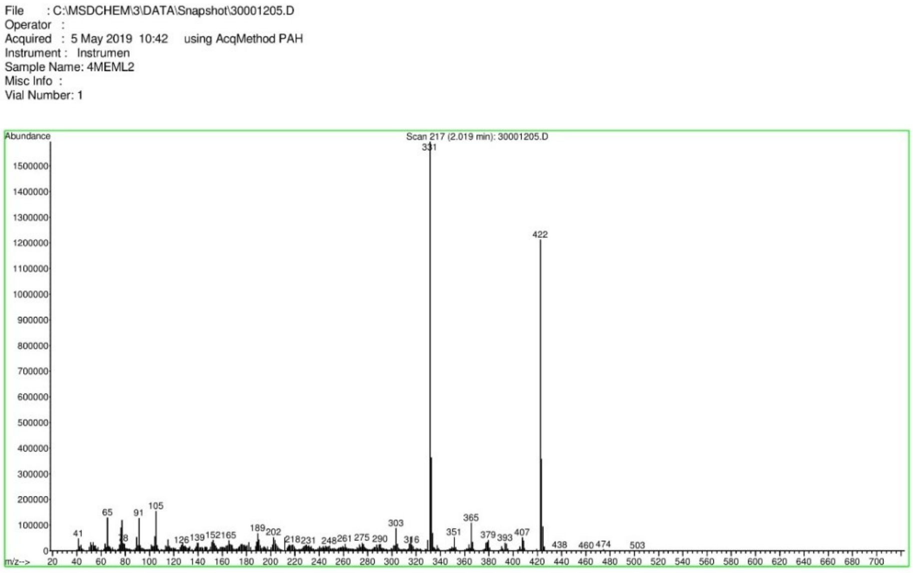

Figure S17. ^{1}H **NMR spectrum of 14-Amino-13-(p-tolyl)-2,3,4,13-tetrahydro-1*H*-benzo[6,7]chromeno[2,3-b]quinoline-7,12-dione**

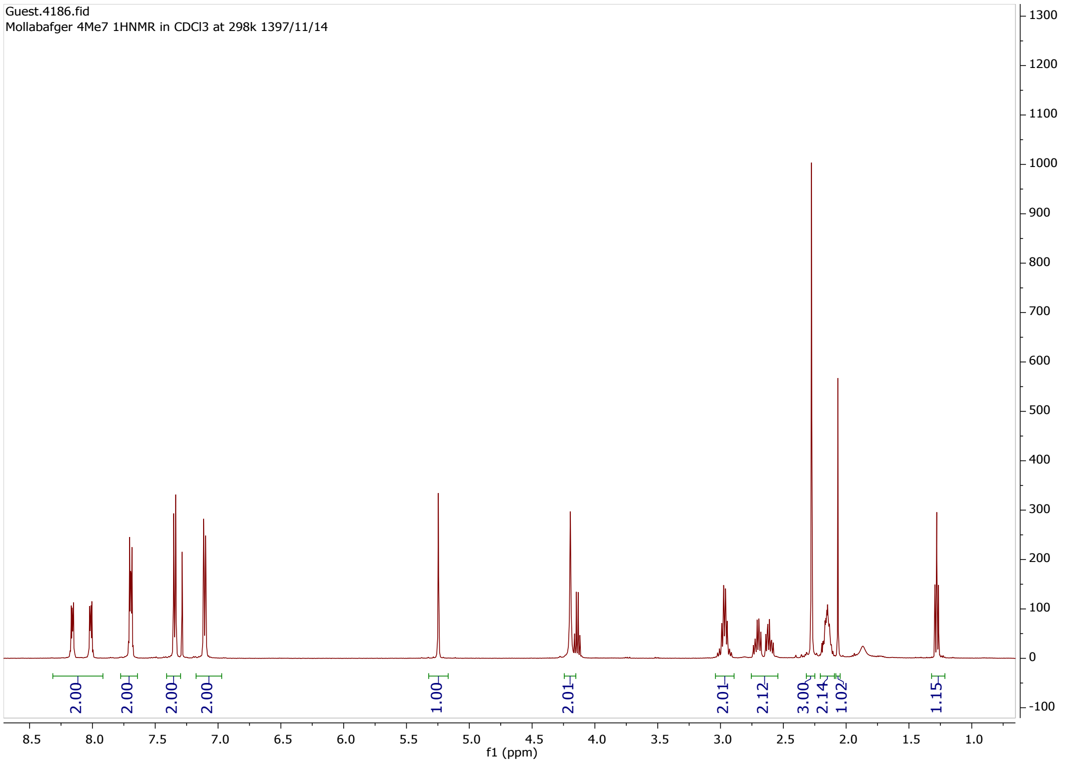

Figure S18. ^{13}C **NMR spectrum of 14-Amino-13-(p-tolyl)-2,3,4,13-tetrahydro-1*H*-benzo[6,7]chromeno[2,3-b]quinoline-7,12-dione**

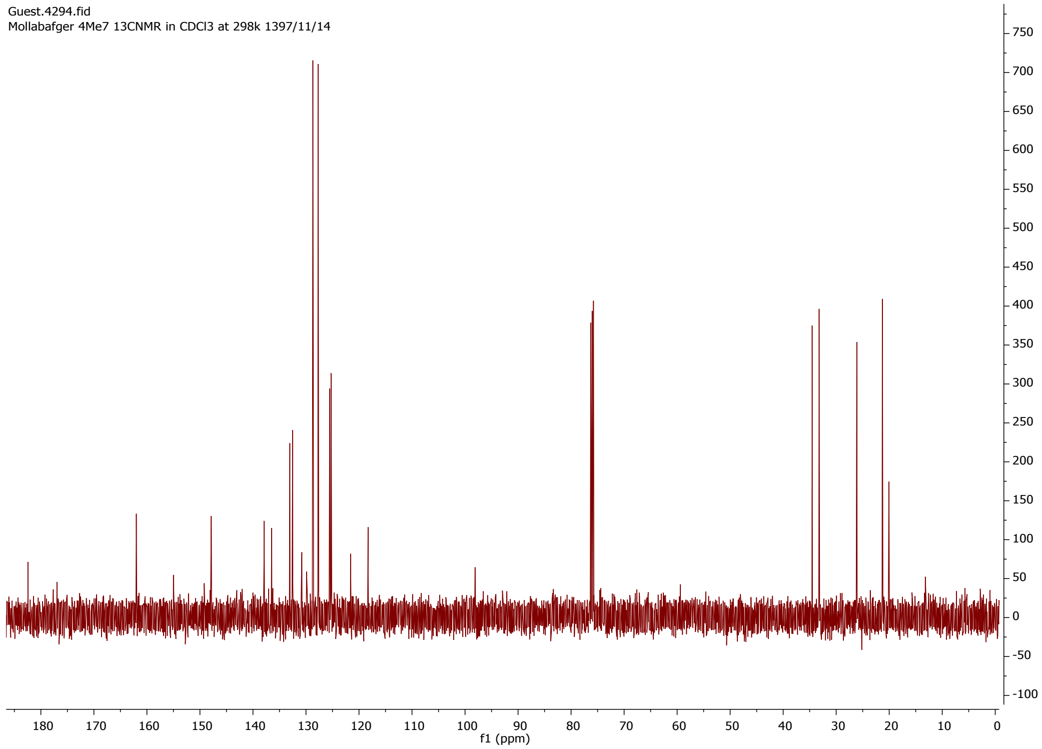

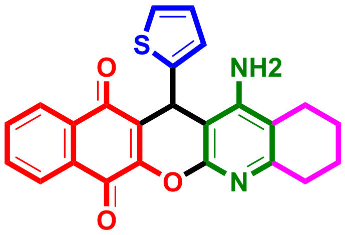

6e

Figure S19. FT-IR spectrum of 14-Amino-13-(thiophen-2-yl)-2,3,4,13-tetrahydro-1*H*-benzo[6,7]chromeno[2,3-b]quinoline-7,12-dione

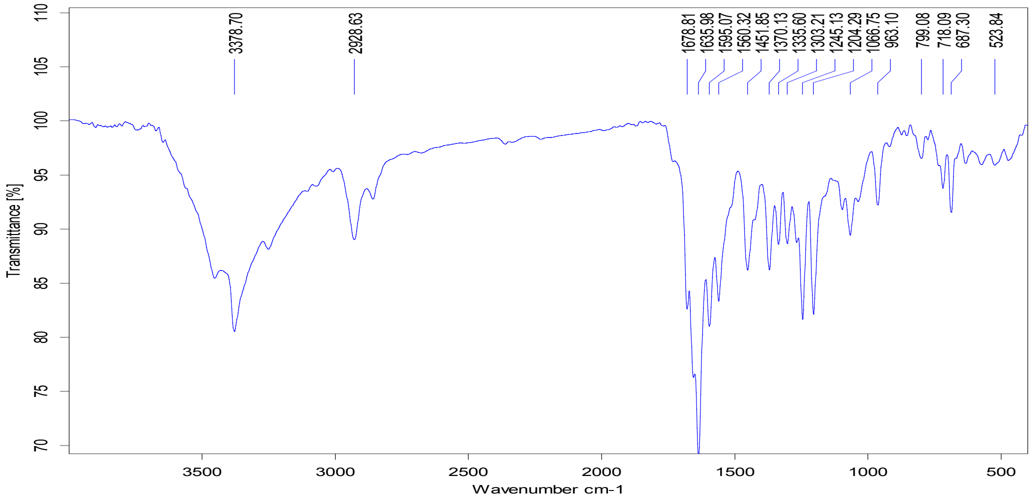

Figure S20. Mass spectrum of 14-Amino-13-(thiophen-2-yl)-2,3,4,13-tetrahydro-1*H*-benzo[6,7]chromeno[2,3-b]quinoline-7,12-dione

File : C:\MSDCHEM\3\DATA\Snapshot\30001206.D
Operator :
Acquired : 5 May 2019 10:54 using AcqMethod PAH
Instrument : Instrumen
Sample Name: THIOML2
Misc Info :
Vial Number: 1

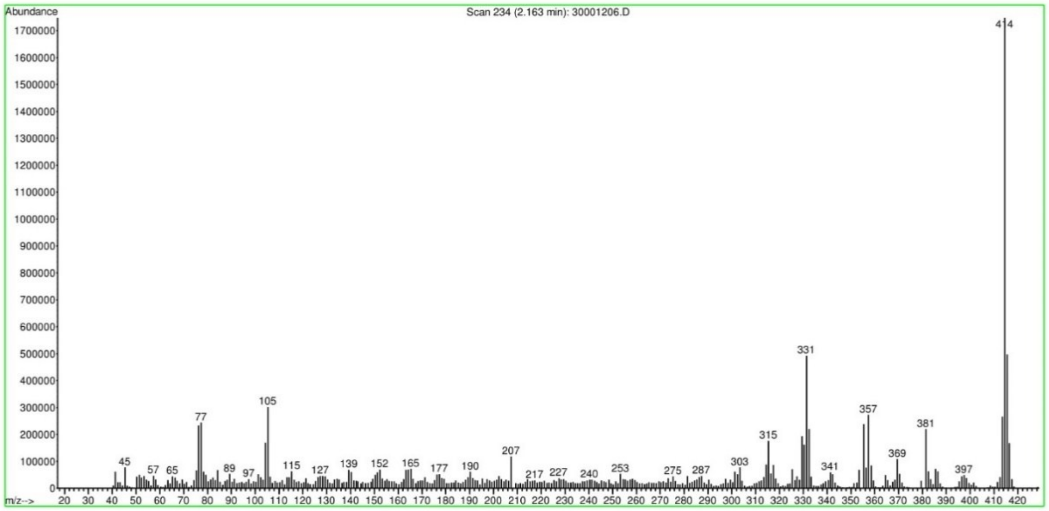

Figure S21. ^{1}**H NMR spectrum of 14-Amino-13-(thiophen-2-yl)-2,3,4,13-tetrahydro-1*H*-benzo[6,7]chromeno[2,3-b]quinoline-7,12-dione**

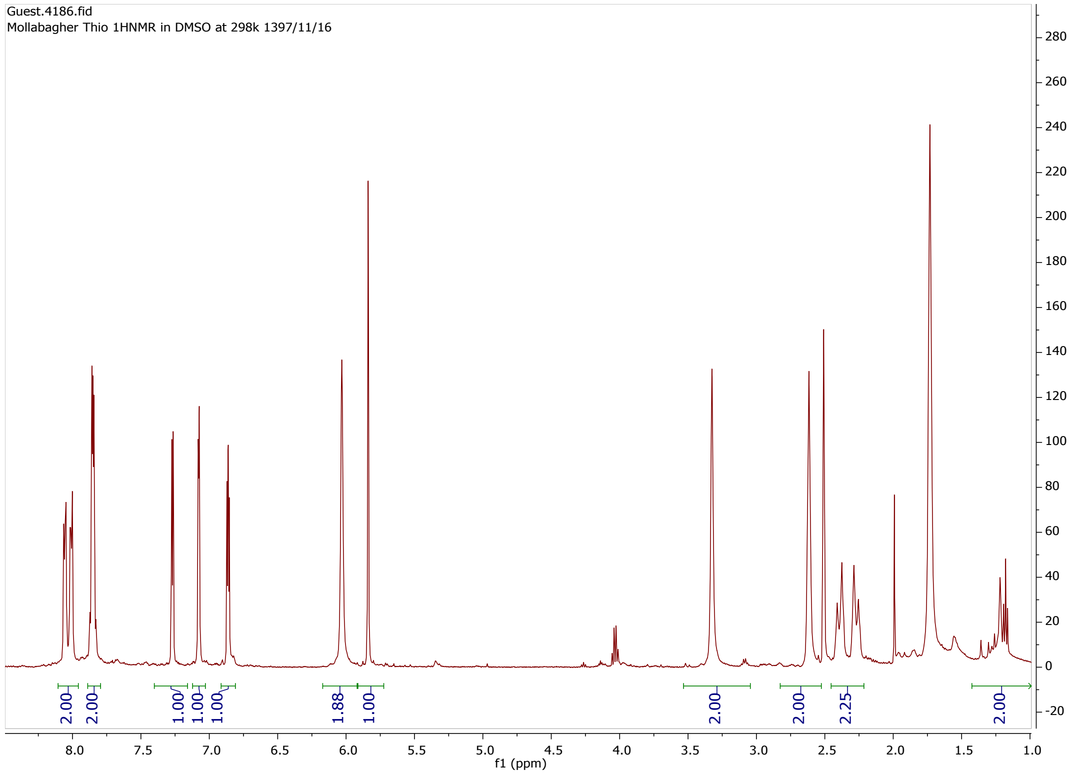

Figure S22. ^{13}**C NMR spectrum of 14-Amino-13-(thiophen-2-yl)-2,3,4,13-tetrahydro-1*H*-benzo[6,7]chromeno[2,3-b]quinoline-7,12-dione**

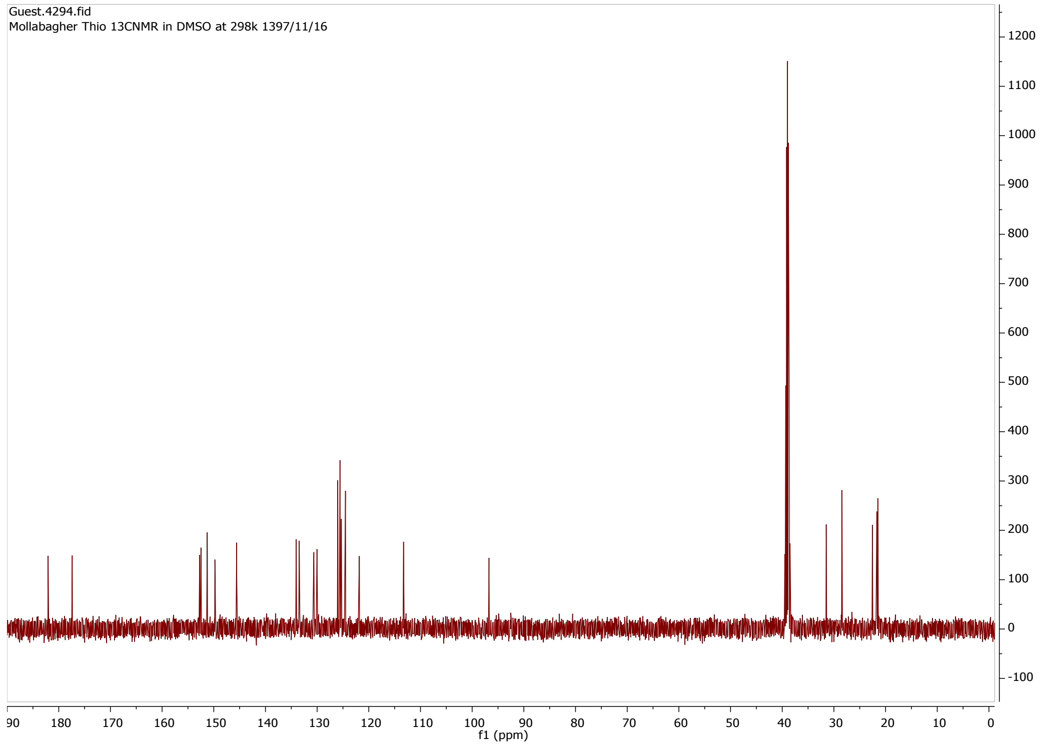

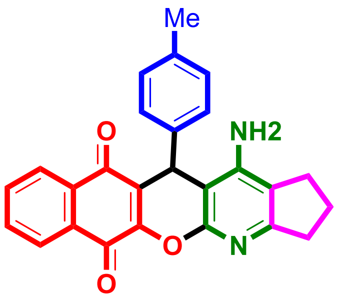

6f

Figure S23. FT-IR spectrum of 13-Amino-12-(p-tolyl)-1,2,3,12-tetrahydrobenzo[6,7]chromeno[2,3-b]cyclopenta[e]pyridine-6,11-dione

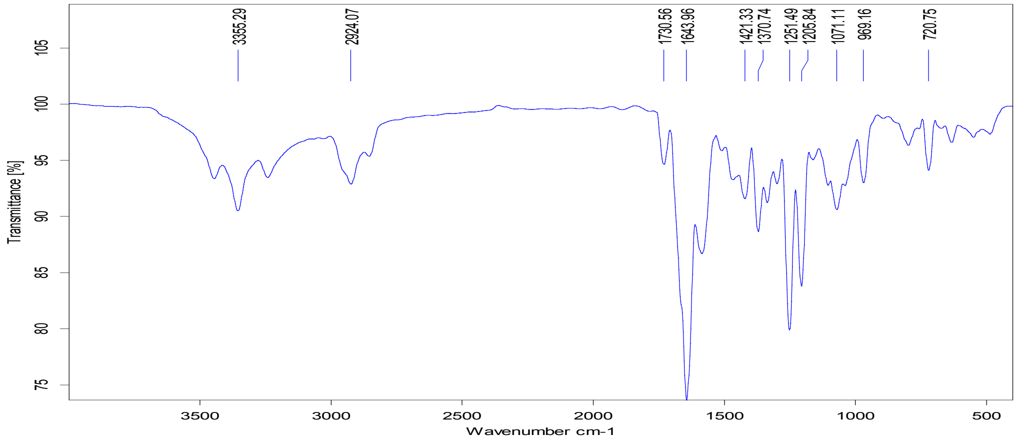

Figure S24. Mass spectrum of 13-Amino-12-(p-tolyl)-1,2,3,12-tetrahydrobenzo[6,7]chromeno[2,3-b]cyclopenta[e]pyridine-6,11-dione

File : C:\MSDCHEM\3\DATA\Snapshot\30001201.D
Operator :
Acquired : 5 May 2019 10:01 using AcqMethod PAH
Instrument : Instrumen
Sample Name: 4MeM5
Misc Info :
Vial Number: 1

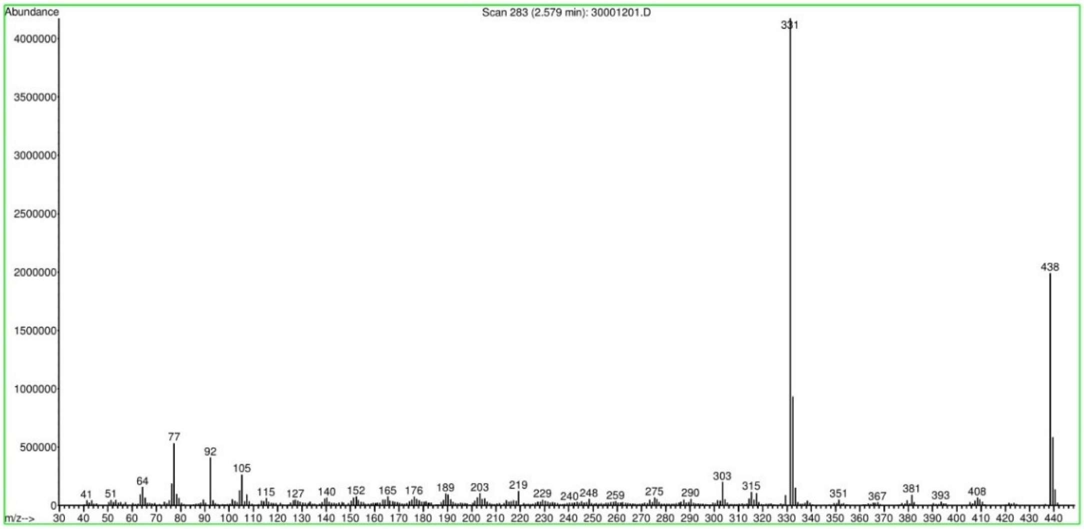

Figure S25. ^{1}H NMR spectrum of 13-Amino-12-(p-tolyl)-1,2,3,12-tetrahydrobenzo[6,7]chromeno[2,3-b]cyclopenta[e]pyridine-6,11-dione

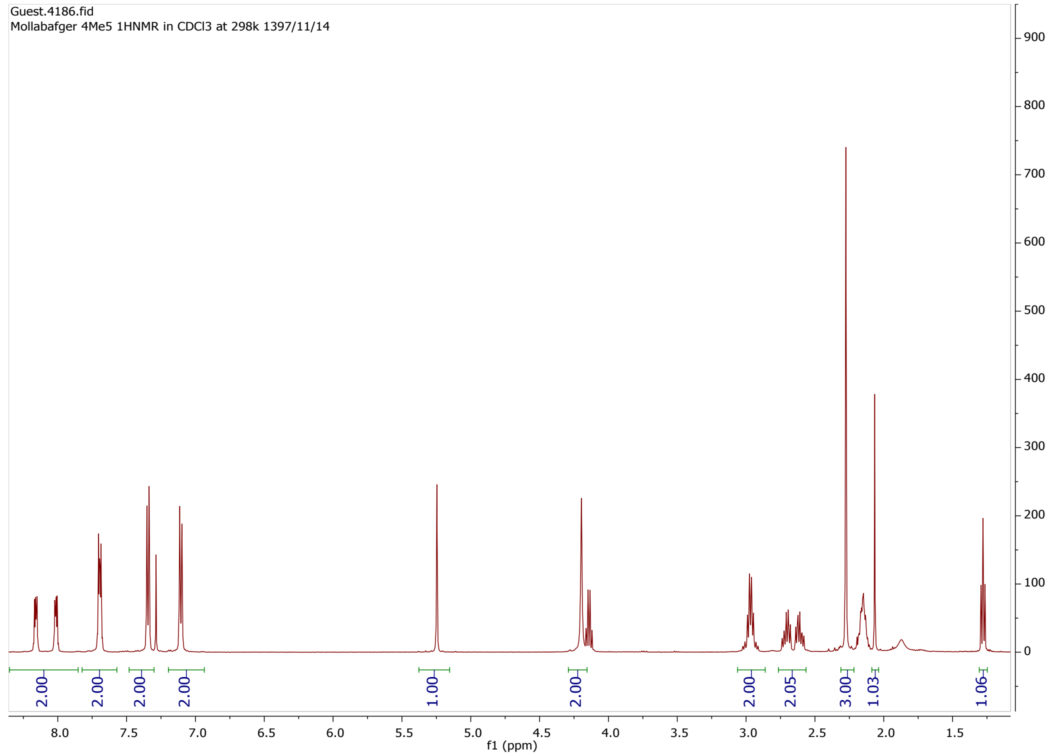

Figure S26. ^{13}C NMR spectrum of 13-Amino-12-(p-tolyl)-1,2,3,12-tetrahydrobenzo[6,7]chromeno[2,3-b]cyclopenta[e]pyridine-6,11-dione

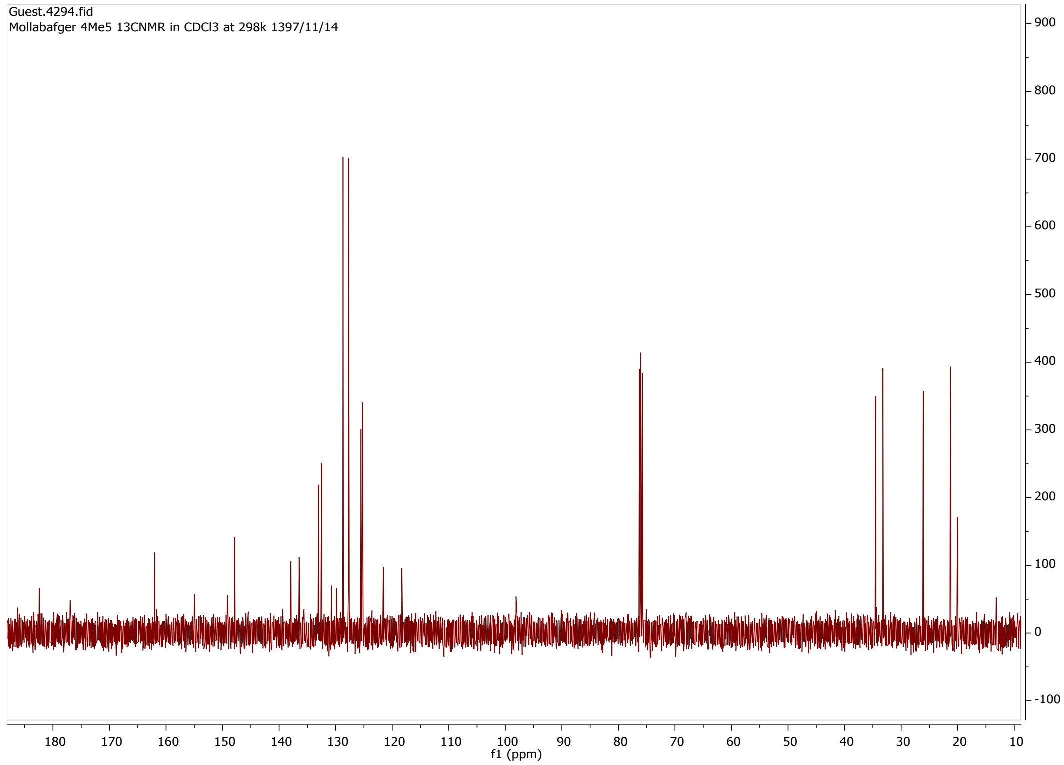

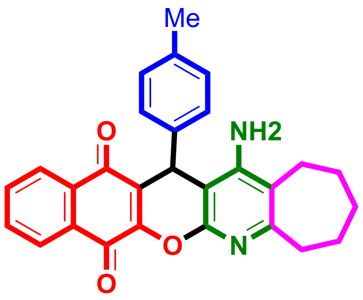

6g

Figure S27. FT-IR spectrum of 13-Amino-14-(p-tolyl)-8,9,10,11,12,14-hexahydrobenzo[6,7]chromeno[2,3 b]cyclohepta[e]pyridine-5,15-dione

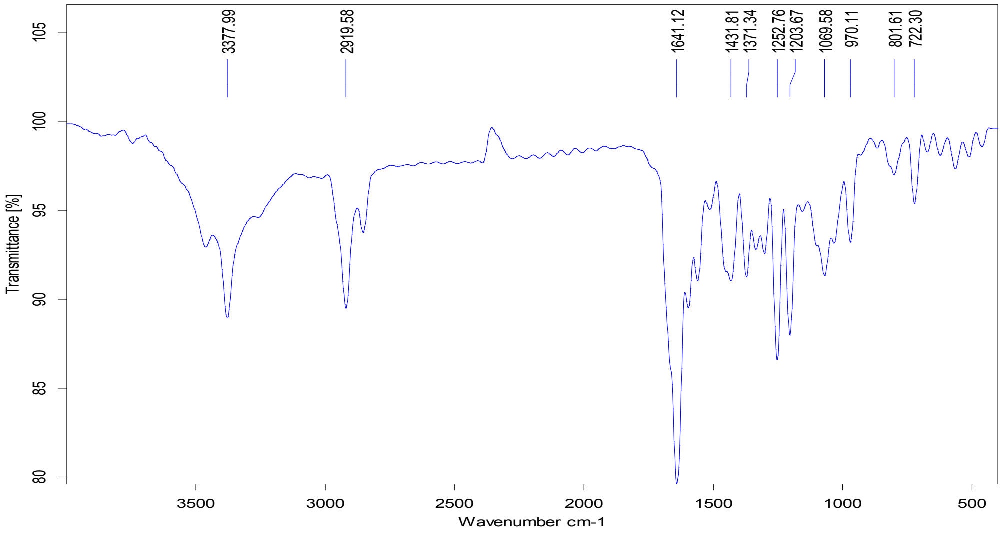

Figure S28. Mass spectrum of 13-Amino-14-(p-tolyl)-8,9,10,11,12,14-hexahydrobenzo[6,7]chromeno[2,3 b]cyclohepta[e]pyridine-5,15-dione

File : C:\MSDCHEM\3\DATA\Snapshot\30001207.D
Operator :
Acquired : 5 May 2019 11:03 using AcqMethod PAH
Instrument : Instrumen
Sample Name: 4MEM7
Misc Info :
Vial Number: 1

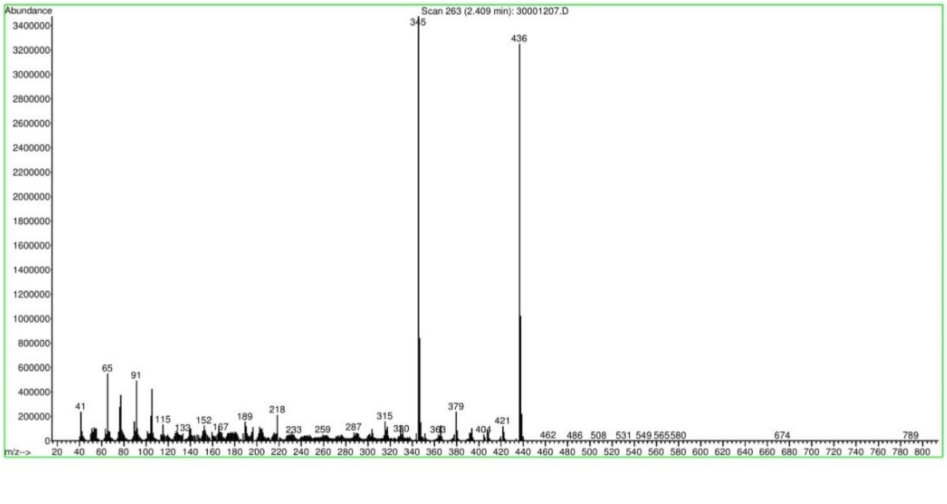

Figure S29. ^{1}H **NMR spectrum of 13-Amino-14-(p-tolyl)-8,9,10,11,12,14-hexahydrobenzo[6,7]chromeno[2,3 b]cyclohepta[e]pyridine-5,15-dione**

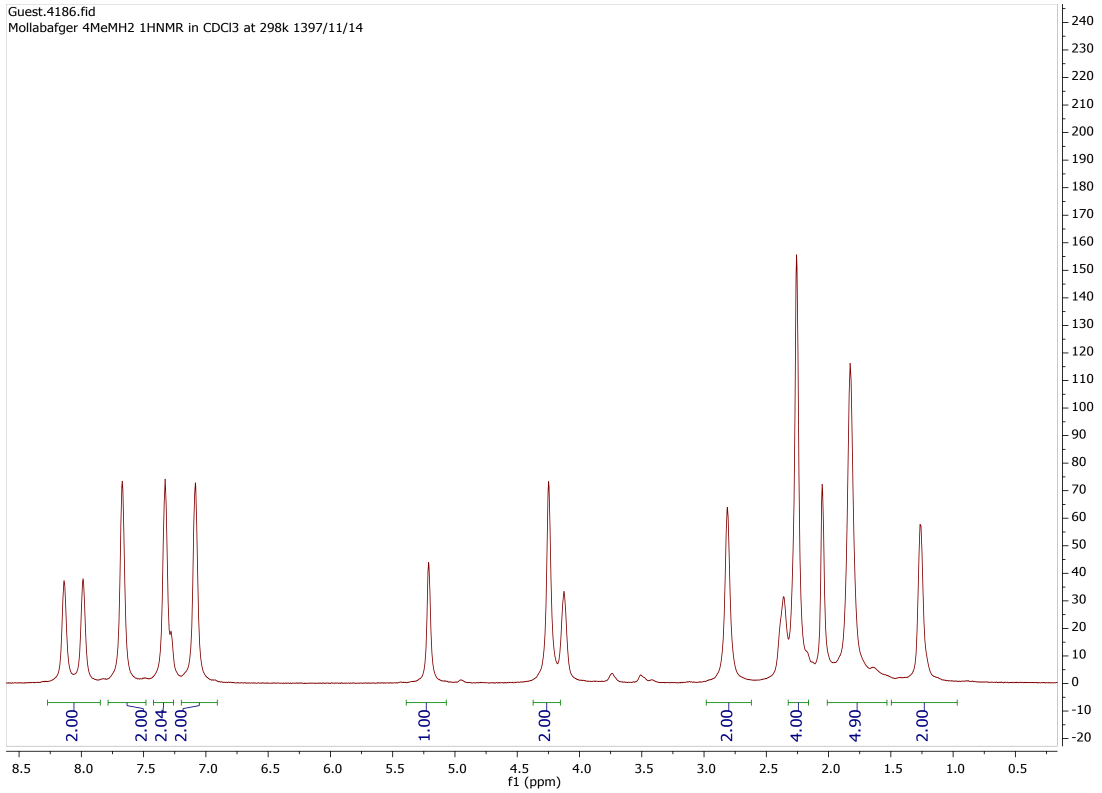

Figure S30. ^{13}C **NMR spectrum of 13-Amino-14-(p-tolyl)-8,9,10,11,12,14-hexahydrobenzo[6,7]chromeno[2,3 b]cyclohepta[e]pyridine-5,15-dione**

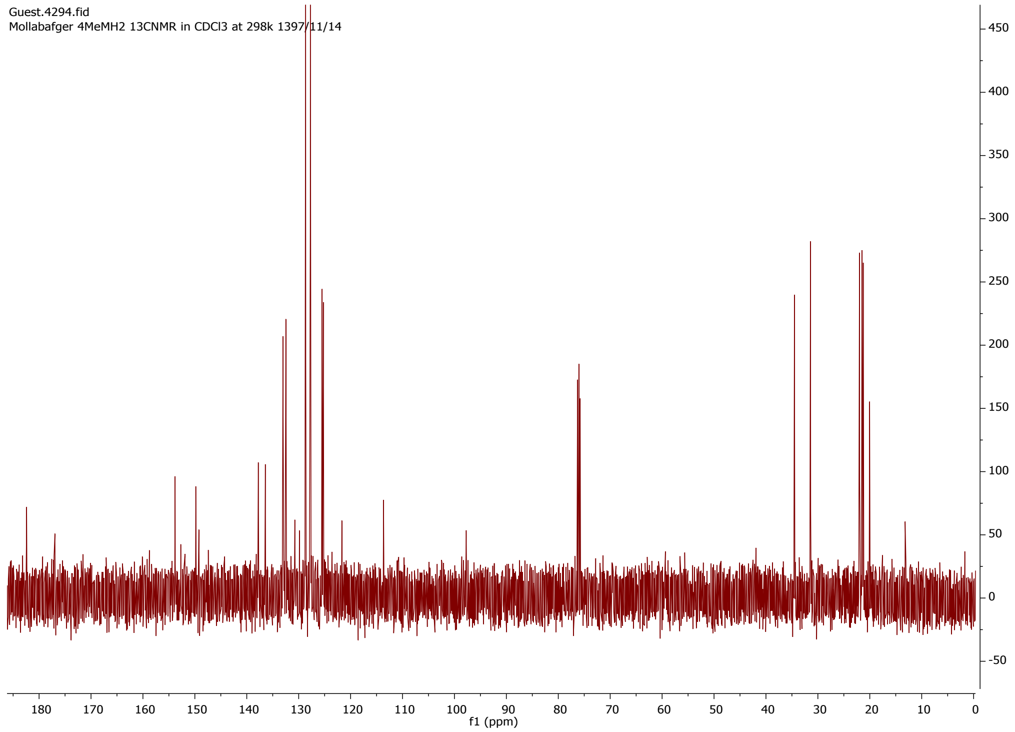

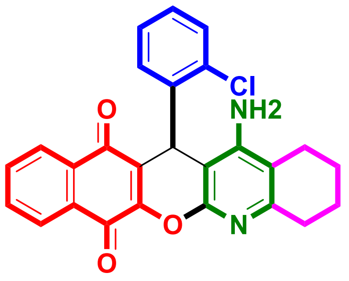

6h

Figure S31. FT-IR spectrum of 14-Amino-13-(2-chlorophenyl)-2,3,4,13-tetrahydro-1H-benzo[6,7]chromeno[2,3-b]quinoline-7,12-dione

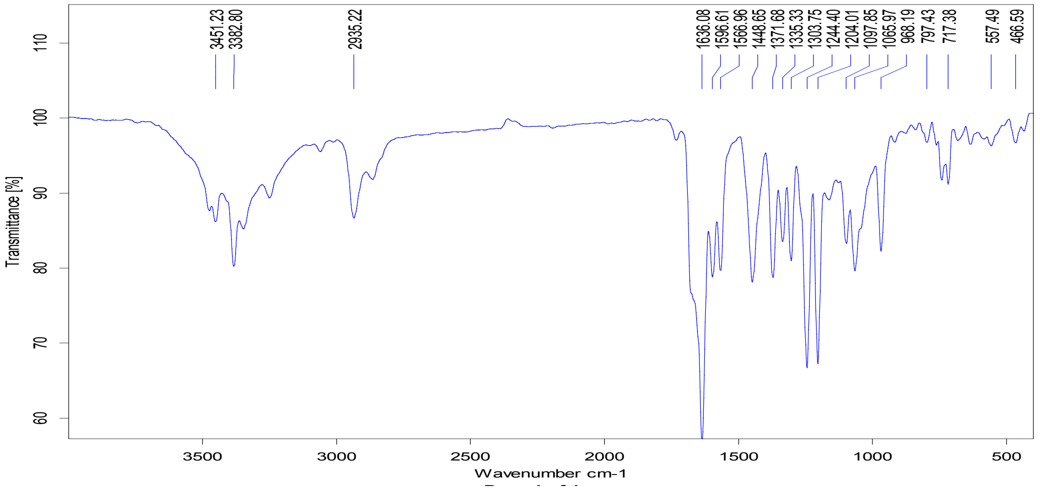

Figure S32. Mass spectrum of 14-Amino-13-(2-chlorophenyl)-2,3,4,13-tetrahydro-1H-benzo[6,7]chromeno[2,3-b]quinoline-7,12-dione

File : C:\MSDCHEM\3\DATA\Snapshot\30001208.D
Operator :
Acquired : 5 May 2019 11:18 using AcqMethod PAH
Instrument : Instrumen
Sample Name: 2CLML2
Misc Info :
Vial Number: 1

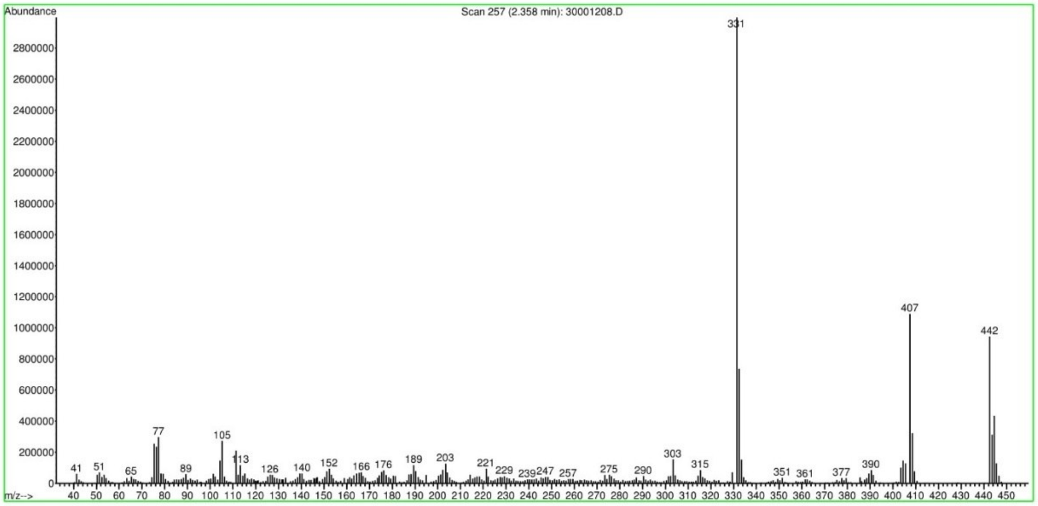

Figure S33. ^{1}H NMR spectrum of 14-Amino-13-(2-chlorophenyl)-2,3,4,13-tetrahydro-1H-benzo[6,7]chromeno[2,3-b]quinoline-7,12-dione

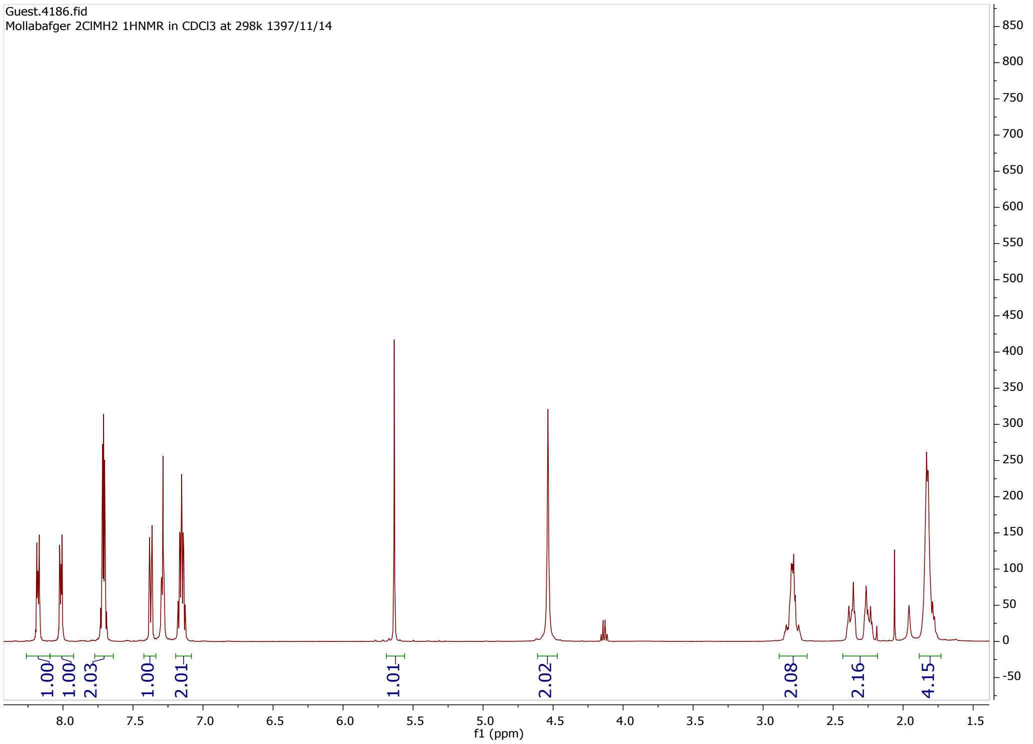

Figure S34. ^{13}C NMR spectrum of 14-Amino-13-(2-chlorophenyl)-2,3,4,13-tetrahydro-1H-benzo[6,7]chromeno[2,3-b]quinoline-7,12-dione

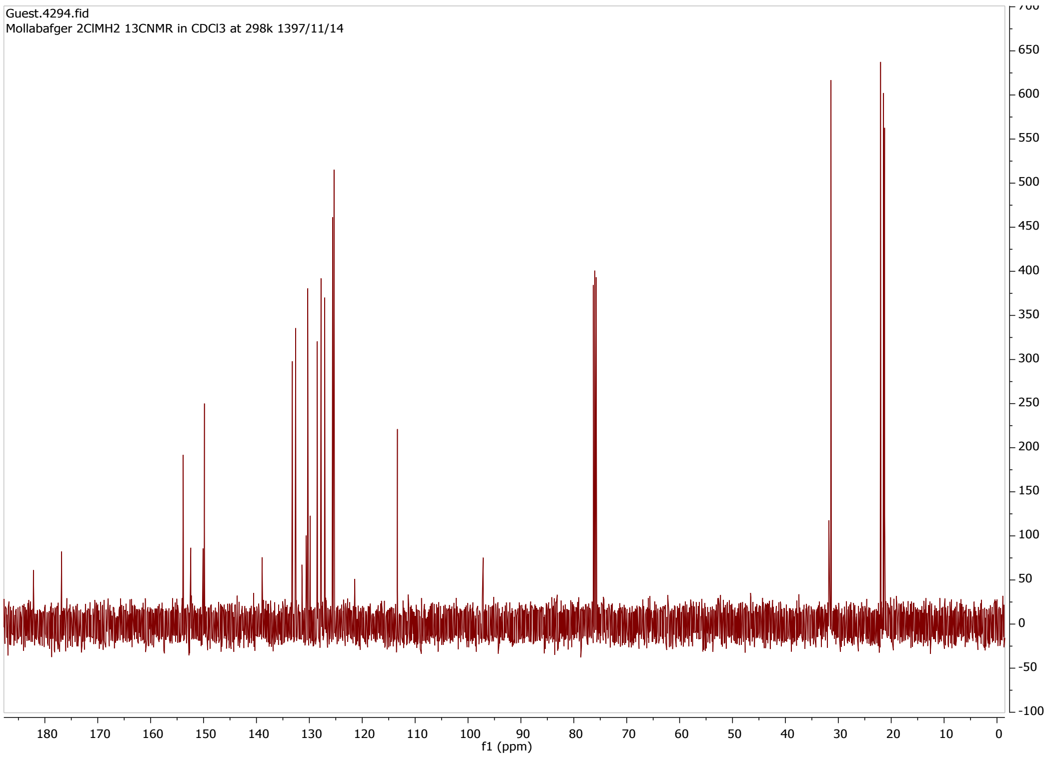

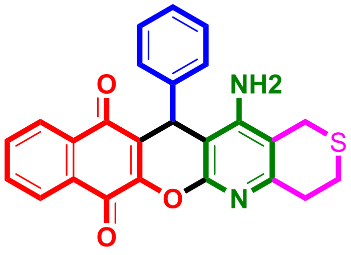

6i

Figure S35. FT-IR spectrum of 14-Amino-13-phenyl-4,13-dihydro-1H,3H-benzo[6,7]chromeno[2,3-b]thiopyrano[3,4-e]pyridine-7,12-dione

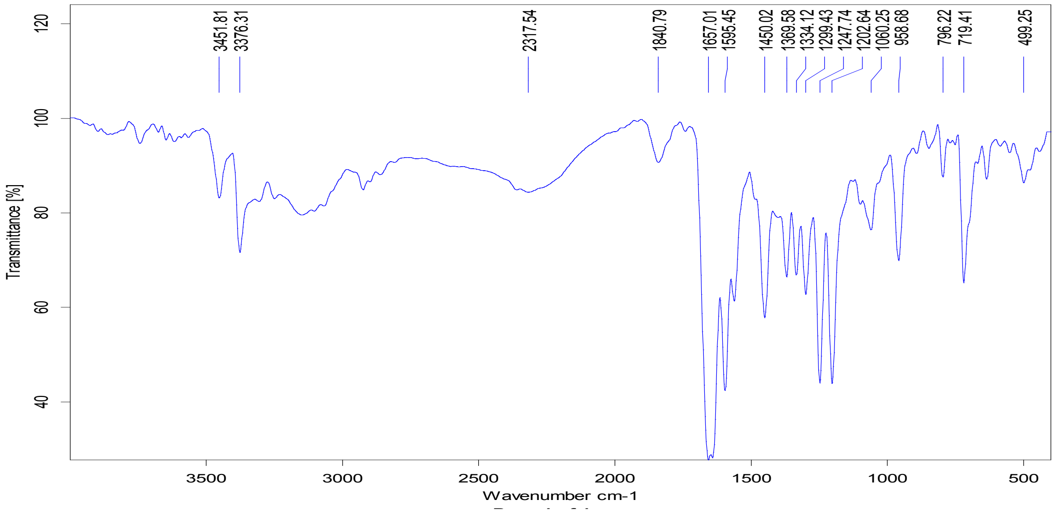

Figure S36. Mass spectrum of 14-Amino-13-phenyl-4,13-dihydro-1H,3H-benzo[6,7]chromeno[2,3-b]thiopyrano[3,4-e]pyridine-7,12-dione

File : C:\MSDCHEM\3\DATA\Snapshot\30001209.D
Operator :
Acquired : 5 May 2019 11:31 using AcqMethod PAH
Instrument : Instrumen
Sample Name: BMLS
Misc Info :
Vial Number: 1

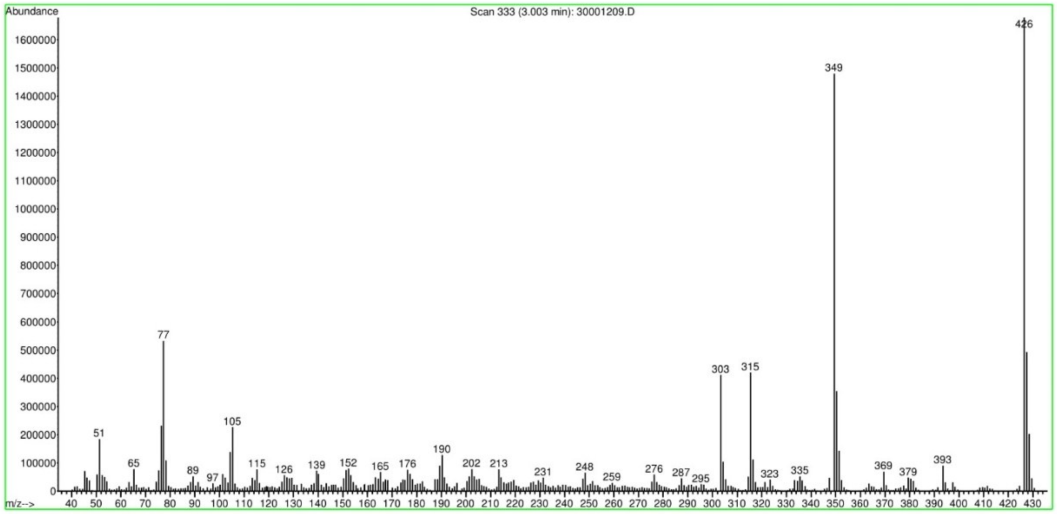

Figure S37. ^{1}H NMR spectrum of 14-Amino-13-phenyl-4,13-dihydro-1H,3H-benzo[6,7]chromeno[2,3-b]thiopyrano[3,4-e]pyridine-7,12-dione

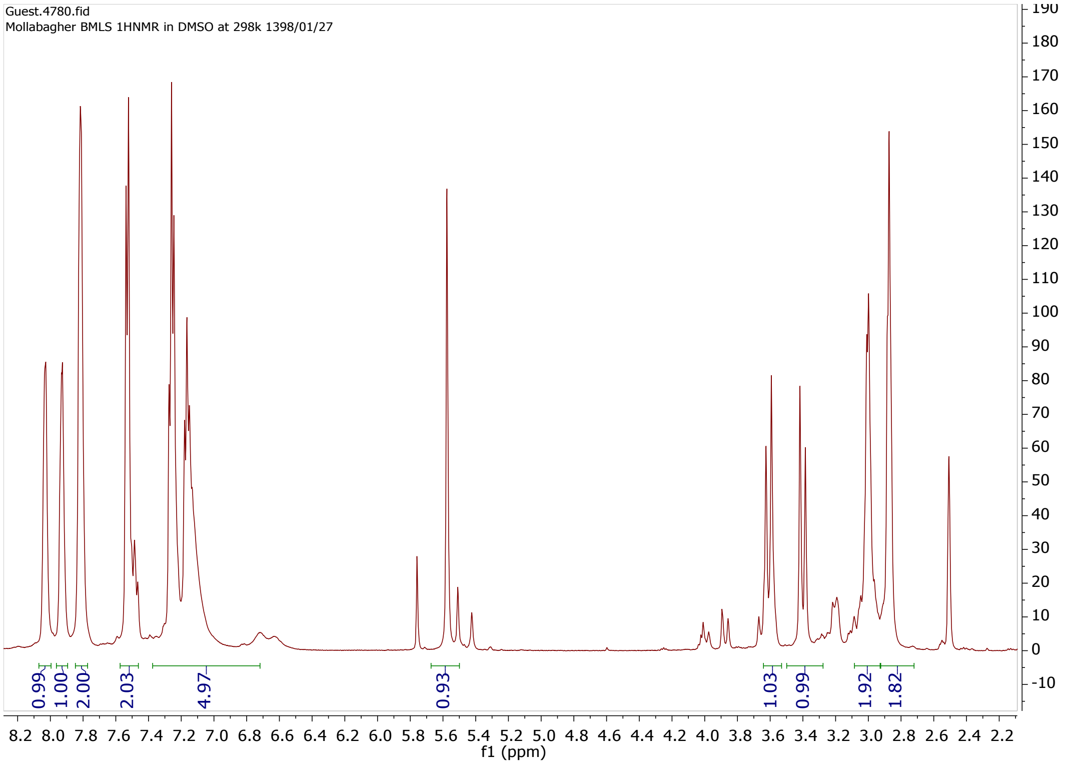

Figure S38. ^{13}C NMR spectrum of 14-Amino-13-phenyl-2,3,4,13-tetrahydro-1H-benzo[6,7]chromeno[2,3-b]quinoline-7,12-dione

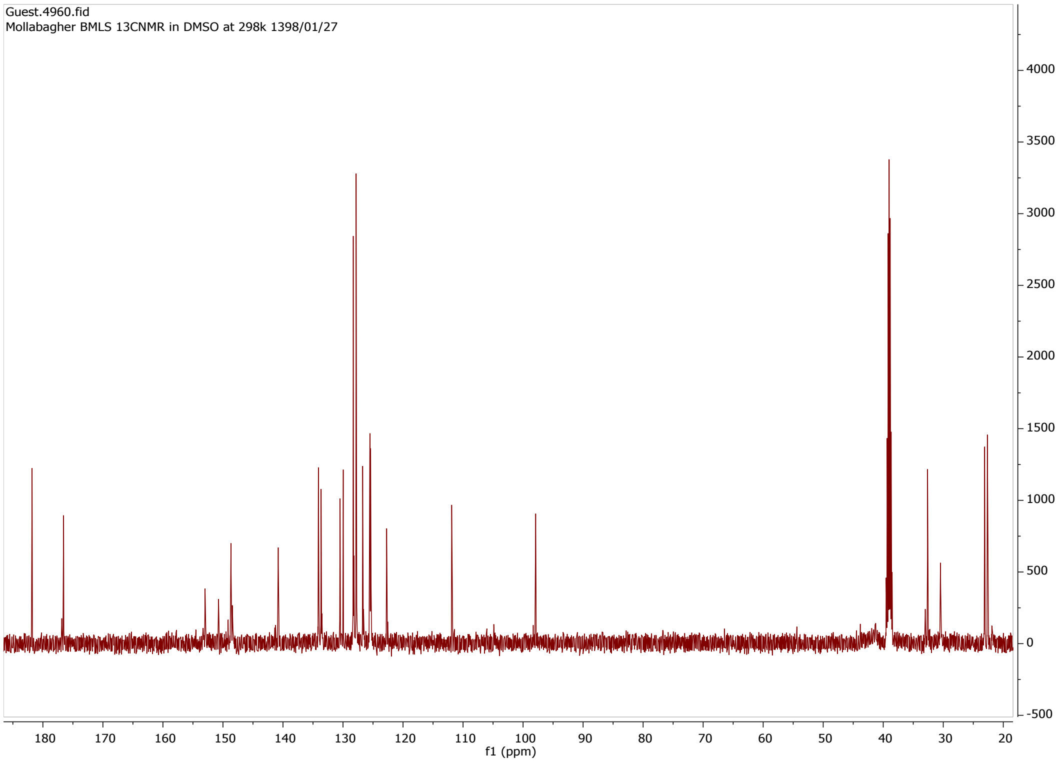

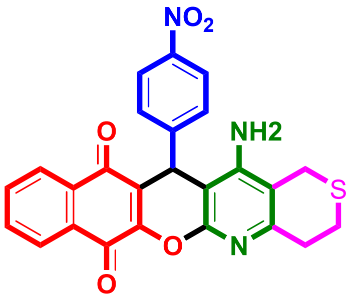

6j

Figure S39. FT-IR spectrum of 14-Amino-13-(4-nitrophenyl)-4,13-dihydro-1H,3H-benzo[6,7]chromeno[2,3-b]thiopyrano[3,4-e]pyridine-7,12-dione

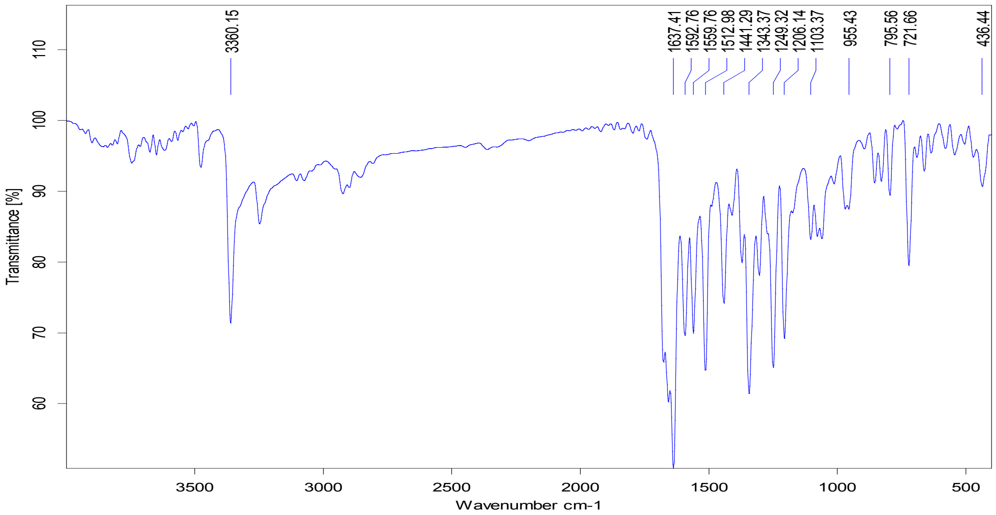

Figure S40. Mass spectrum of 14-Amino-13-(4-nitrophenyl)-4,13-dihydro-1H,3H-benzo[6,7]chromeno[2,3-b]thiopyrano[3,4-e]pyridine-7,12-dione

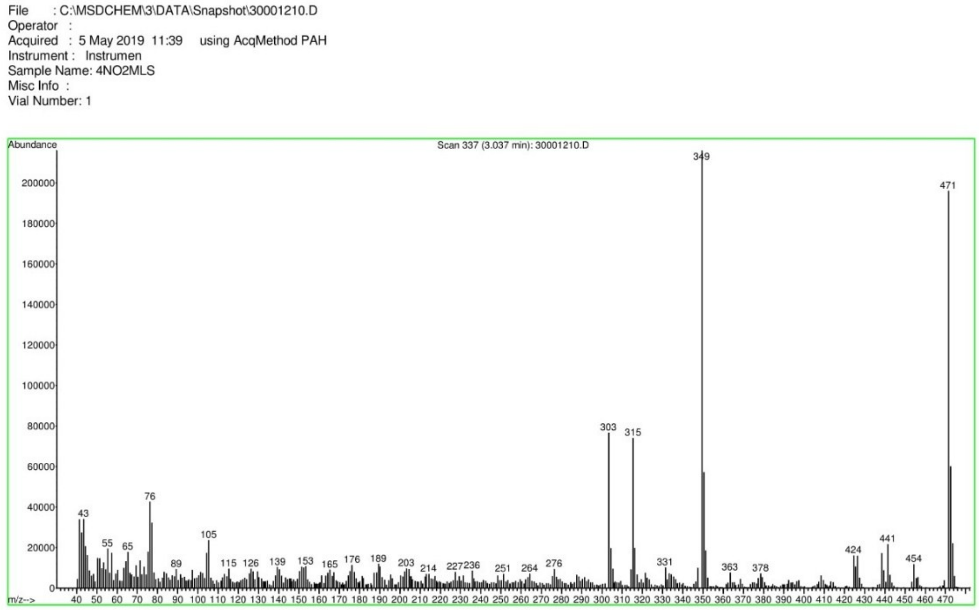

Figure S41. ^{1}H **NMR spectrum of 14-Amino-13-(4-nitrophenyl)-4,13-dihydro-1H,3H-benzo[6,7]chromeno[2,3-b]thiopyrano[3,4-e]pyridine-7,12-dione**

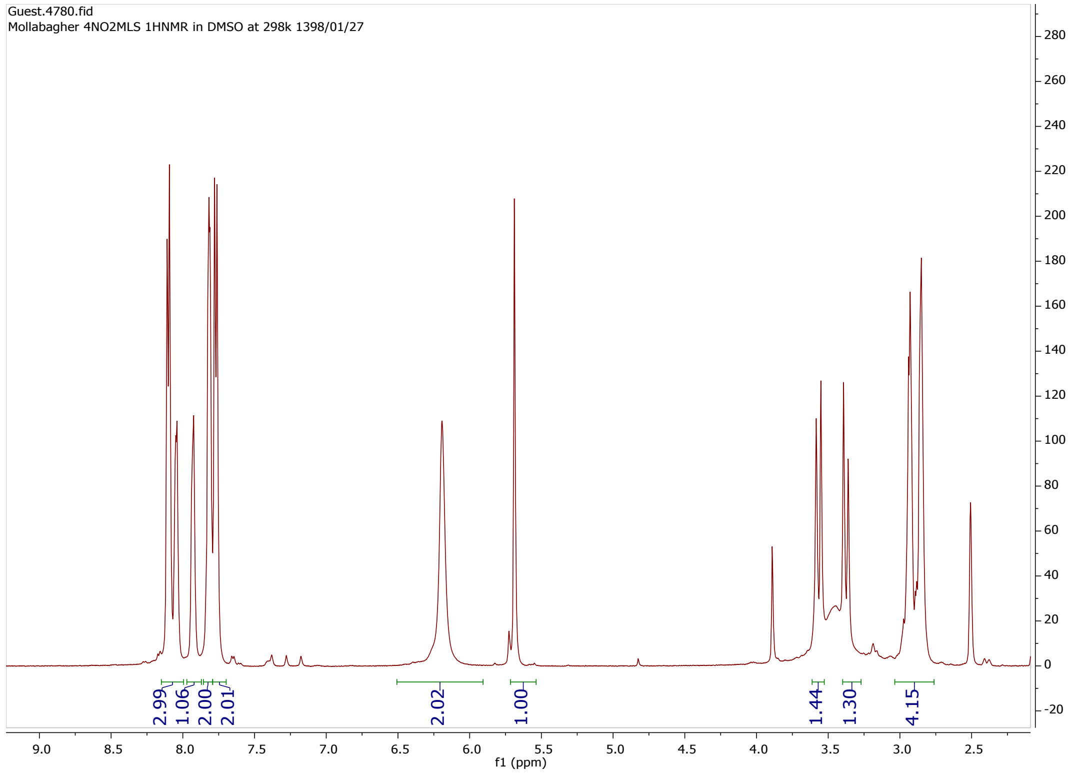

Figure S42. ^{13}C **NMR spectrum of 14-Amino-13-(4-nitrophenyl)-4,13-dihydro-1H,3H-benzo[6,7]chromeno[2,3-b]thiopyrano[3,4-e]pyridine-7,12-dione**

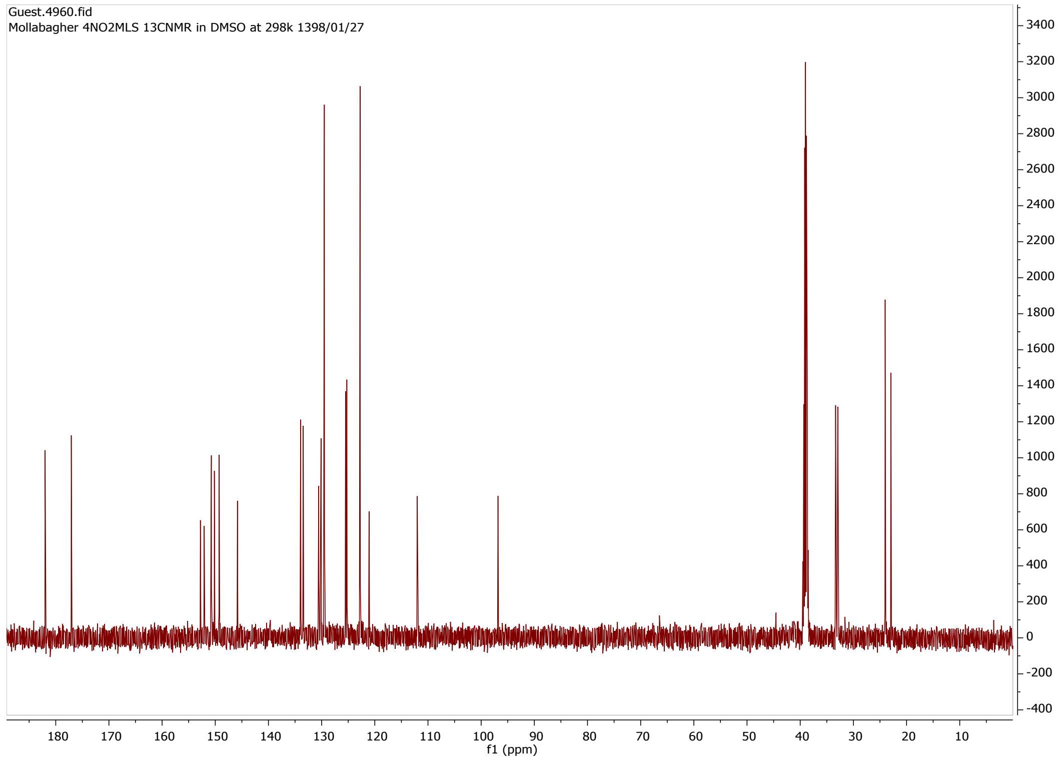

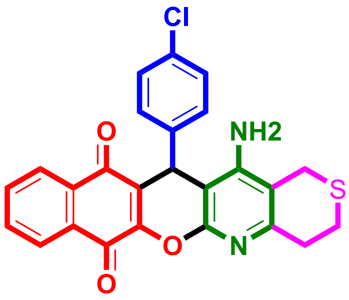

6k

Figure S43. FT-IR spectrum of 14-Amino-13-(4-chlorophenyl)-4,13-dihydro-1H,3H-benzo[6,7]chromeno[2,3-b]thiopyrano[3,4-e]pyridine-7,12-dione

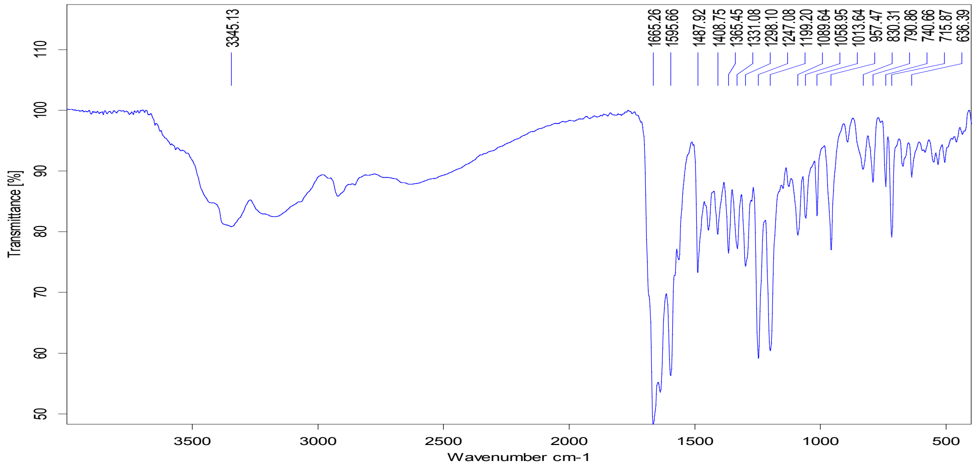

Figure S44. Mass spectrum of 14-Amino-13-(4-chlorophenyl)-4,13-dihydro-1H,3H-benzo[6,7]chromeno[2,3-b]thiopyrano[3,4-e]pyridine-7,12-dione

File : C:\MSDCHEM\3\DATA\Snapshot\30001211.D
Operator :
Acquired : 5 May 2019 11:50 using AcqMethod PAH
Instrument : Instrumen
Sample Name: 4CLMLS
Misc Info :
Vial Number: 1

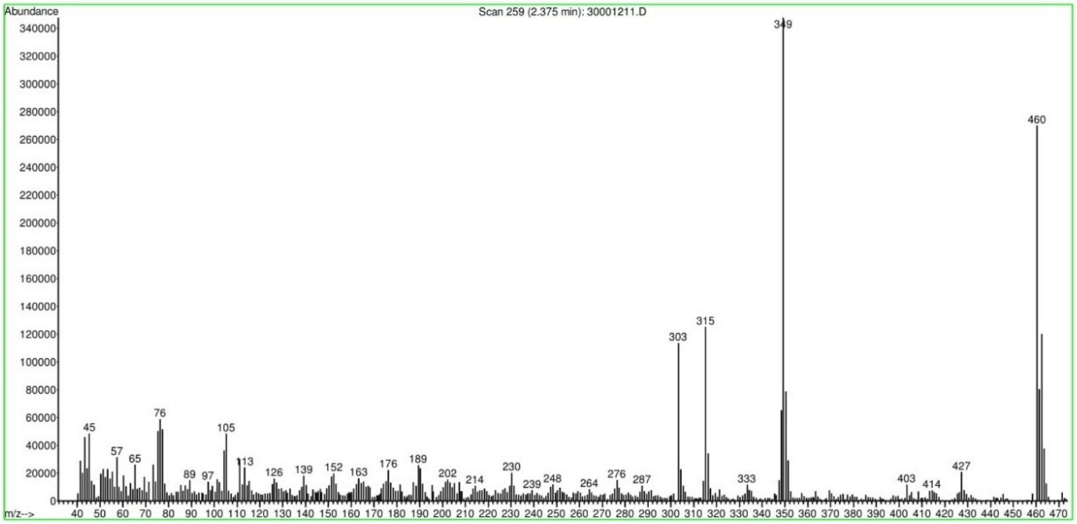

Figure S45. ^{1}H NMR spectrum of 14-Amino-13-(4-chlorophenyl)-4,13-dihydro-1H,3H-benzo[6,7]chromeno[2,3-b]thiopyrano[3,4-e]pyridine-7,12-dione

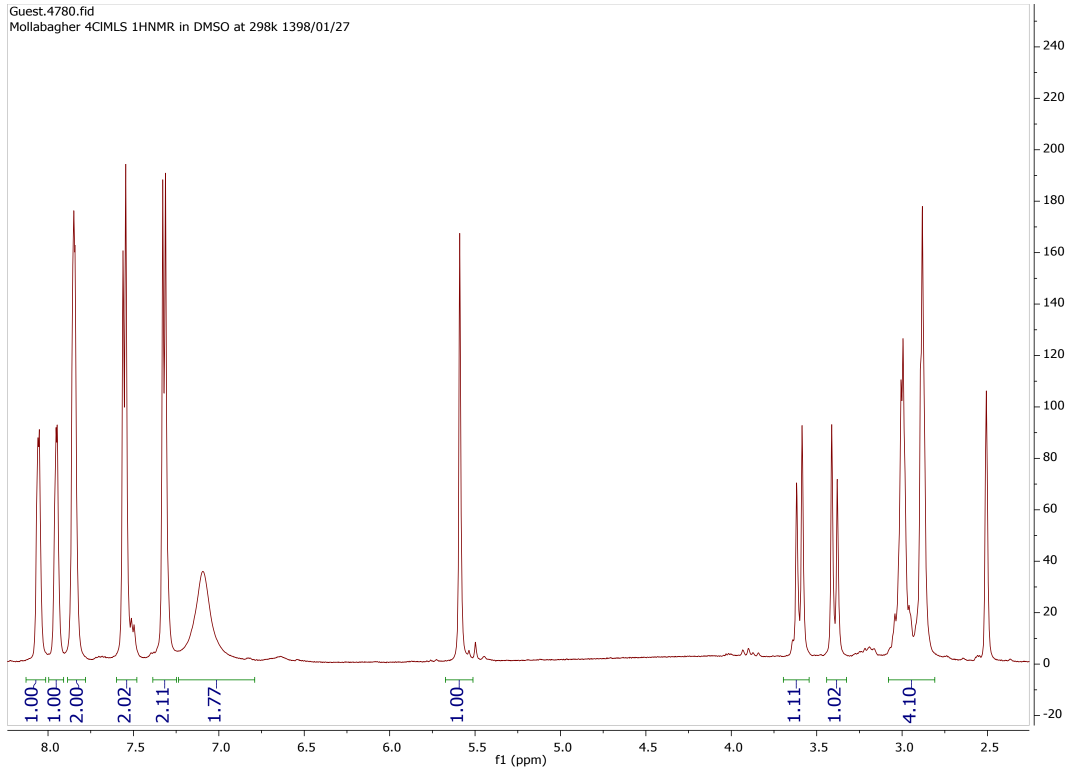

Figure S46. ^{13}C NMR spectrum of 14-Amino-13-(4-chlorophenyl)-4,13-dihydro-1H,3H-benzo[6,7]chromeno[2,3-b]thiopyrano[3,4-e]pyridine-7,12-dione

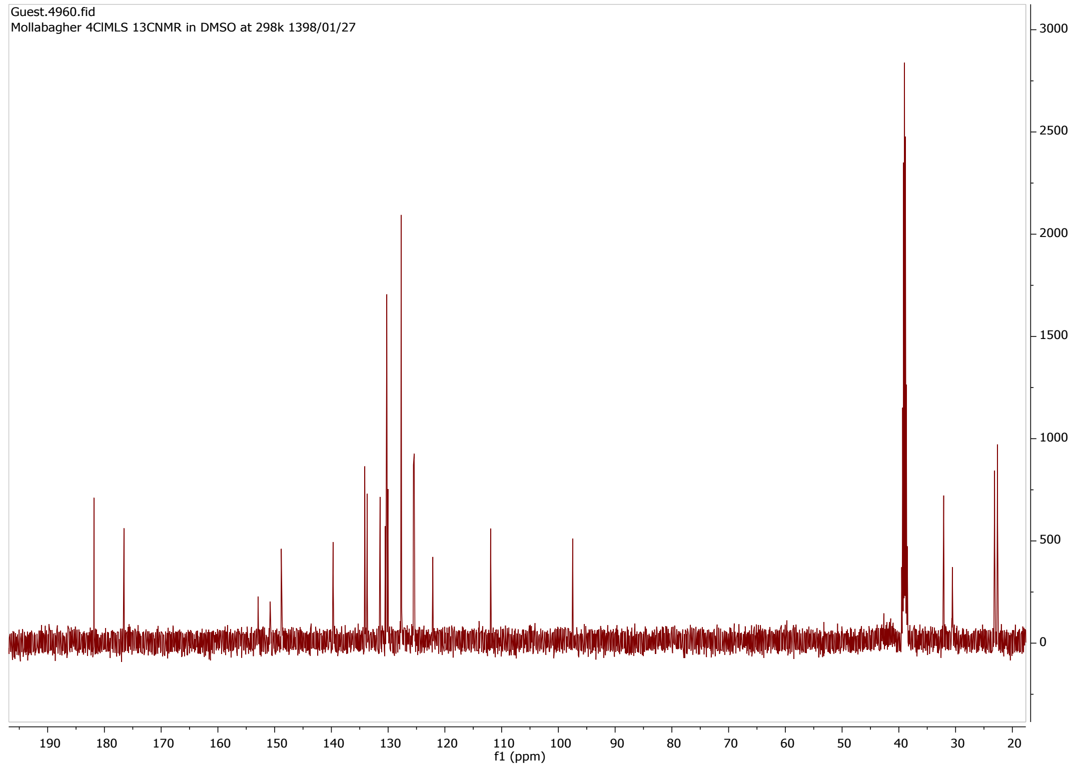

Supplement: RA-010-C9RA10111J-s001 [file RA-010-C9RA10111J-s001.pdf]
